# High intensity forced ultrasound-driven ferroptosis as a strategy for anti-tumor immune priming

**DOI:** 10.1016/j.apsb.2025.05.006

**Published:** 2025-05-28

**Authors:** Xuejing Li, Jiayi Wu, Ruizhe Xu, Xifeng Qin, Siyu Wang, Wuli Yang, Zhiqing Pang

**Affiliations:** a Key Laboratory of Smart Drug Delivery, School of Pharmacy, Fudan University, Shanghai 201203, China; b State Key Laboratory of Molecular Engineering of Polymers & Department of Macromolecular Science, Fudan University, Shanghai 200433, China

**Keywords:** High intensity forced ultrasound, Perfluorooctyl bromide, Ferroptosis, GSH/GSSG balance, Triple-negative breast cancer, Tumor microenvironment, Tumor immunotherapy, Nano-enhancer

## Abstract

Cold tumors have a poor response to tumor immunotherapy due to low immune cell infiltration and the ability to evade immune attacks. Converting cold tumors into hot tumors can enhance the clinical effectiveness of anti-tumor immunotherapy. High-intensity focused ultrasound (HIFU) as a non-invasive treatment can damage tumors through mechanical effects, but there is a lack of research on its cytotoxic mechanisms at the cellular level and its role in inducing anti-immune responses. In this study, the role of HIFU in triggering tumor ferroptosis by disrupting the GSH/GSSG balance through mechanochemical action and the associated anti-tumor immune priming effect were investigated. The use of a nano-enhancer loaded with PFOB combined with HIFU could enhance ferroptosis in triple-negative breast cancer at a specific stage of tumor growth (UTGR = 0) while promoting the conversion of a cold tumor into a hot tumor, thereby improving the immune response. Overall, this provides valuable guidance for the clinical application of HIFU in tumor immunotherapy.

## Graphical abstract

The nano-enhancer was injected into tumor-bearing mice at certain tumor growth stage. HIFU irradiation caused GSH/GSSG imbalance and triggered cell ferroptosis, activating anti-tumor immune responses, and leading to tumor regression.

**Figure:**
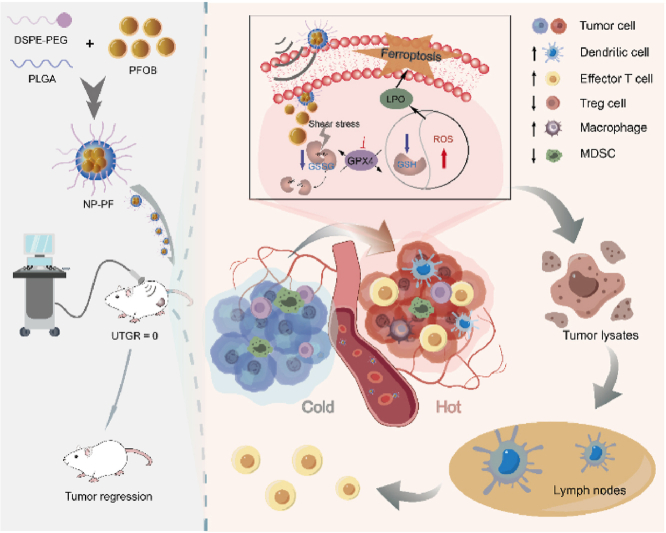

## 1 Introduction

Tumor immunotherapy is a rapidly advancing field that aims to disrupt tumor cells by modulating the immune system at the tumor site^1^. While clinical cases demonstrate impressive inhibition rates, the responsiveness of individual tumor patients to immunotherapy exhibits considerable variation^2^. Tumors are categorized into hot (immune-inflamed) tumors and cold (immune-desert) tumors based on the infiltration level of T lymphocytes and other immune cells in the tumor microenvironment (TME)^3^. Cold tumors show significant potential for evading anti-tumor immunity, making them less responsive to recent immunotherapeutic advances^4^^,^^5^. Various studies propose strategies for turning cold tumors hot such as enhancing tumor cell immunogenicity, optimizing dendritic cell antigen presentation capabilities, bolstering the recognition and cytotoxicity of effector immune cells, and reprogramming the immunosuppressive tumor microenvironment^6^^,^^7^. These approaches are expected to activate antitumor immune responses in combination with immune checkpoints and have limited efficacy in early clinical trials^3^^,^^6^. Hence, novel strategies are urgently required to reshape the tumor microenvironment of cold tumors and increase immune cell infiltration to improve the efficacy of related immunotherapy^8^^,^^9^.

Ultrasound ablation represents a minimally invasive approach for tumor ablation, harnessing thermal and mechanical effects generated by a focused acoustic energy ultrasound probe^10^. High-intensity focused ultrasound (HIFU) exerts damage on tumor cells and large tissues through shear stresses induced by complex bubble oscillation and bubble–tissue–cell interactions^11^. Despite the promising outcomes reported in published studies, clinical experiences reveal rapid energy attenuation with penetration depth. Challenges such as low accuracy and tumor recurrence, stemming from unablated residual tumor tissues, present limitations for broader clinical applications^12^. To enhance therapeutic efficacy, strategies such as optimizing acoustic parameters, including intensified HIFU power, have been considered. However, excessive energy may introduce waveform shock, posing the risk of unintentional adverse effects on normal tissues^13^. Another promising avenue involves the introduction of HIFU enhancement agents, such as microbubbles, which have been found to be applicable in clinical and medical settings^14^. Microbubbles leverage inertial acoustic cavitation to lower the pressure threshold and enhance the acoustic environment for HIFU therapy at tumor sites. Nonetheless, their overlarge size and short lifespan currently hinder their effectiveness and clinical applicability in HIFU therapy^15^.

Preliminary evidence has suggested that HIFU treatment could induce the immune effects in cancer by destroying cancer cells, releasing tumor-specific antigens, providing more stimuli for DC maturation, and allowing more immune therapeutics to access tumors^16^, ^17^, ^18^. However, the underlying mechanism behind HIFU's ability to trigger immunogenic cell death (ICD) is still unclear. Further valuable studies are needed to explore how to elicit and enhance the anti-tumor systemic immune response of HIFU treatment^19^. Recent advancements in the field of mechanochemistry have revealed that ultrasound can induce the scission of mechanochemically labile bonds, such as disulfide bridges, in polymers and biomacromolecules^20^^,^^21^. The disruption of disulfide bonds within proteins plays a crucial role in altering their structure and function^22^. This capability suggests that HIFU can selectively disrupt the disulfide bonds in proteins or peptides such as glutathione oxidized (GSSG), thereby affecting the GSH metabolism pathway and sensitizing cancer cells to ferroptosis. Ferroptosis is a novel mode of regulated cell death owning unique characteristics, such as cell volume shrinkage, decreased mitochondria cristae, and condensed mitochondrial membrane, which was regulated by cellular metabolic events and triggered by iron-dependent accumulation of lipid peroxides^23^^,^^24^. This type of regulated cell death is considered to be inflammatory due to the ability to release pro-inflammatory intracellular contents^25^^,^^26^. Current evidence suggests that ferroptosis plays a role in T cell immunity and cancer immunotherapy, and triggering ferroptosis to modulate the TME represents a tumor cell-extrinsic strategy for overcoming immunotherapy resistance^27^. The induction of cytotoxic effects in response to external stimuli *via* ferroptotic programs is pivotal in anticancer treatments, enhancing the sensitivity of cancer cells to therapeutic interventions^28^. Therefore, exploring the ferroptosis capability of HIFU is of great value in extending its clinical application in tumor immunotherapy.

**Scheme 1:**
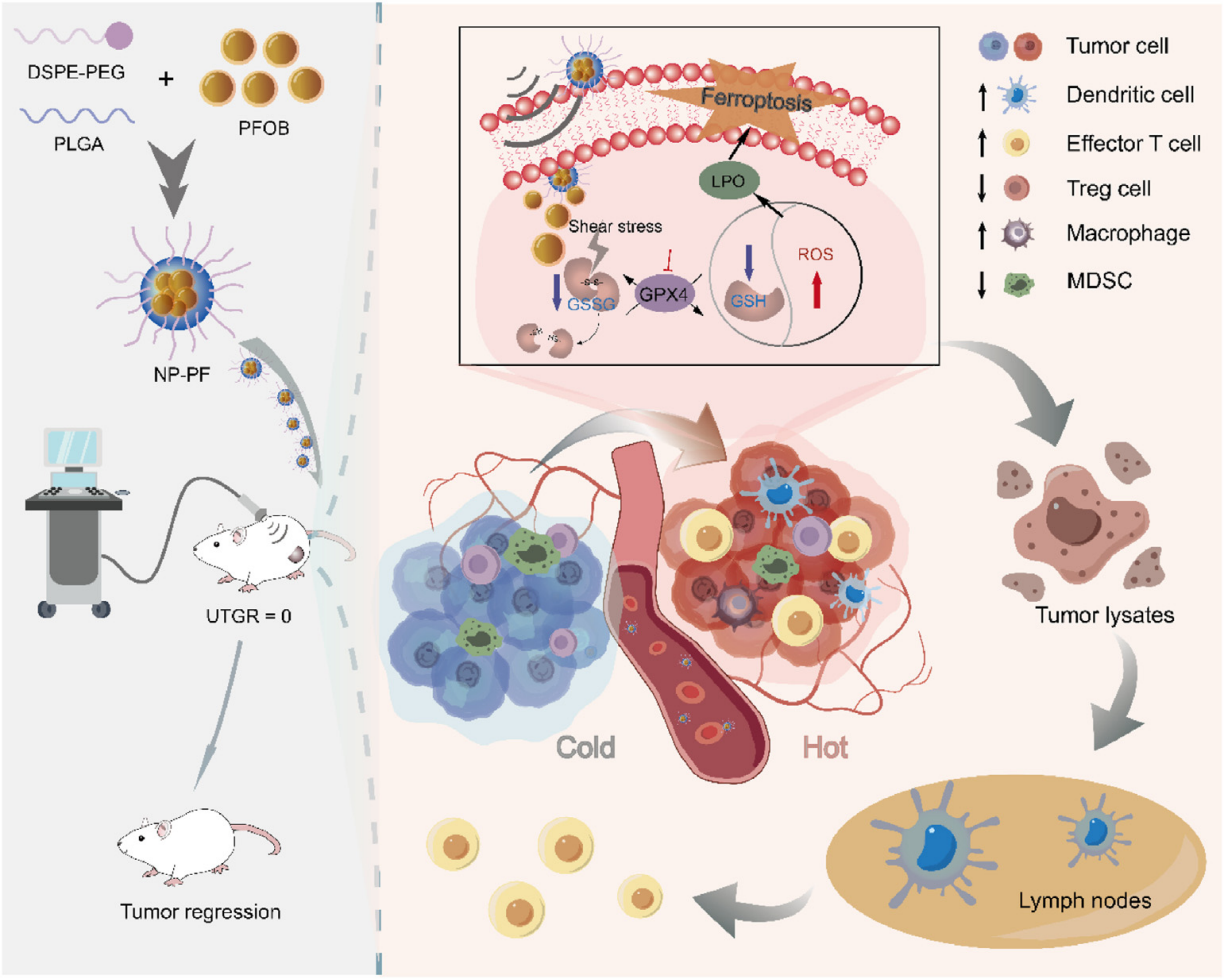
NP-PF-enhanced HIFU for tumor ferroptosis and immune activation in 4T1 tumor-bearing mice.

Herein, in the present study, we validated the role of HIFU in inducing ferroptosis and explored the technical requirements for triggering ferroptosis. As triple-negative breast cancer (TNBC) is a superficial malignant tumor that is quite suitable for HIFU treatment and demonstrates a low level of immune response towards immunotherapy^29^^,^^30^, TNBC was opted as the disease model. Furthermore, we developed nano-enhancers to enhance the shear stress of HIFU. Coordinated treatment at the appropriate stage of tumor growth aims to increase the sensitivity of tumor cells to ferroptosis induced by HIFU therapy, subsequently enhancing anti-tumor immune responses (Scheme 1). Perfluorooctyl bromide (PFOB), an Food and Drug Administration (FDA)-approved dense liquid with a biocompatible nature and a low diffusion coefficient into blood, was chosen to construct nano-enhancers with a long lifespan^31^. In addition, the underlying mechanism of HIFU to trigger ferroptosis through mechanochemical action and the associated immune priming effect was investigated in depth. Therefore, the combined treatment of HIFU and nano-sensitizers at a specific stage of tumor growth (UTGR = 0) could achieve the full coverage of tumor tissues efficiently and kill primary tumors. More importantly, nano-enhancers could potentiate the ferroptotic effects of HIFU by strengthening the mechanochemical action of HIFU on intracellular GSSG, promoting the transformation of cold tumors into hot tumors and thereby triggering a more robust immune response (Scheme 1).

## 2 Materials and methods

### 2.1 Materials

Hydroxyl-terminated poly(lactic-*co*-glycolic acid) (PLGA-OH; 0.67 dL/g, 50:50 ratio) was purchased from Lactel (Arab, USA). 1,2-Distearoyl-*sn*-glycero-3-phosphoethanolamine-*N*-[methoxy(polyethylene glycol)-2000] (DSPE-PEG2000) was purchased from Laysan Bio Co. (Arab, USA). Perfluorooctyl bromide (PFOB), 2-(4-amidinophenyl)-6-indolecarbamidine dihydrochloride (DAPI), the Cell Counting Kit-8 (CCK8), Annexin V-FITC/PI Cell Apoptosis Kit, Calcein-AM/PI staining kit, 2,7-dichlorofuorescin diacetate (DCFH-DA), and the GSH/GSSG assay kit were purchased from Beyotime Biotechnology (Shanghai, China). Perfluoro-15-crown-5-ether and deuterochloroform (CDCl_3_) were purchased from Sigma (Missouri, USA). BODIPY™581/591 C11 was from Invitrogen (Carlsbad, USA). Ferrostain-1 (Fer-1), deferoxamine (DFO), and vitamin E (VE) were purchased from MedChemExpress (Shanghai, China). RPMI-1640 cell culture medium, granulocyte-macrophage colony-stimulating factor (GM-CSF), interleukin-4 (IL-4), fetal bovine serum (FBS), penicillin-streptomycin (PS), Trypsin–EDTA (0.25%), and 1 × PBS (7.4) were all purchased from Gibco (Waltham, USA). TNF-*α* ELISA kit and IFN-*γ* ELISA kit were from Multisciences (Hangzhou, China). 4% paraformaldehyde was from Biosharp (Anhui, China). Anti-CD11c-FITC, anti-CD80-PE, anti-CD86-PE/Cy7, anti-CD3-AF700, anti-CD4-FITC, anti-CD8-PE, anti-CD45-BV510, anti-CD25-APC, anti-CD11b-PE/Cy7, anti-Gr-1-APC, anti-CD45-APC, anti-F4/80-AF488, anti-CD86-PE, anti-CD206-PerCP-Cy5.5, anti-CD8-FITC, anti-CD44-PE, anti-CD62L-APC, and anti-FOXP3-PE were purchased from BioLegend (California, USA). Anti-GPX4 antibody and anti-PTGS2 antibody were purchased from Abcam (Cambridge, UK).

The 4T1 cell line was purchased from American Type Culture Collection (ATCC, USA), cultured in 1640 basic medium supplemented with 10% FBS and 1% penicillin/streptomycin, and maintained at 37 °C in a 5% CO_2_ environment. CT26 and TC-1 cell lines were obtained from ATCC (USA). The BALB/c mice of 18–20 g were purchased at the Shanghai SLAC Laboratory Animal Ltd. (Shanghai, China). Mouse models of breast cancer were established by subcutaneous injection of 5 × 10^6^ 4T1 cells into the right leg of each mouse. All animal experiments were performed according to the protocol approved by the Ethics Committee of Fudan University (Approval number: 2022-03-YJ-PZQ-08).

### 2.2 Ex vivo ablative capabilities of HIFU

The pork liver models were prepared in degassed water^32^. Pork livers were exposed to HIFU irradiation at different power, including 2.8, 8.5, 10.1, and 11.6 W irradiated for 10, 30, 50, and 70 s, respectively. The cross-sectional digital images of the treated pork livers cut along the direction of HIFU irradiation were recorded and the ablation width and depth were measured. The ablation volume was calculated using Eq. (1):

(1) $$\text{Volume}=W^{2}\times D/2$$

where *W* means width and *D* means depth.

The formula for calculating the energy efficiency factor (EEF) was as in Eq. (2) ^33^:

(2) EEF=η×P×T/V

where *η* = 0.7, *P* means power, *T* means time, *V* means volume.

### 2.3 Validation of cell ferroptosis induced by HIFU

#### 2.3.1 Apoptosis assay of tumor cells after HIFU treatment

The assessment of apoptosis in 4T1 cells treated with HIFU was conducted using the Annexin V-FITC/PI Cell Apoptosis Kit following the manufacturer's guidelines. In brief, 4T1 cells underwent HIFU treatment with different powers (2.8, 8.5, 10.1, and 11.6 W) for different times (10, 30, 50, and 70 s) respectively, and were cultured for 24 h. Subsequently, cells were collected through trypsin digestion and centrifugation, and then stained with Annexin V-FITC and propidium iodide (PI) for 20 min at room temperature in a dark environment. The stained cells were analyzed using a FACS Calibur flow cytometer (BD Biosciences, East Rutherford, USA) and FlowJo v.7.6.5 software (Tree Star, Inc., Ashland, USA) was used for flow cytometry analysis.

#### 2.3.2 Lipid peroxidation (LPO) production in tumor cells after HIFU treatment

4T1 cells were seeded onto 12-well chamber slides and cultured overnight. Subsequently, the cells underwent HIFU irradiation (2.8, 8.5, 10.1, and 11.6 W were irradiated for 10, 30, 50, and 70 s, respectively) and were further cultured for 1 h. Following this, the cells were incubated with C11-BODIPY at 37 °C for 30 min, followed by three washes with PBS. The fluorescence signal of LPO in the cells was analyzed with a FACS Calibur flow cytometer (BD Biosciences, East Rutherford, USA). In addition, LPO signals (stained with C11-BODIPY) in 4 T1 cells after 8.5 W HIFU treatment group (including 0, 10, 30, 50, and 70 s) were visualized using a CLSM (Zeiss, Germany). To determine LPO generation in other cells after HIFU treatment, both CT26 cells and TC1 cells were seeded, cultured, treated with 8.5 W HIFU for 50 s, stained with C11-BODIPY, and analyzed with flow cytometer as described above.

#### 2.3.3 Cell viability of tumor cells treated with HIFU in the presence of ferroptosis inhibitors

To validate the ferroptosis of 4T1 cells treated with HIFU, cell viability of 4T1 cells was tested after HIFU treatment in the presence of ferroptosis inhibitors. Briefly, 4T1 cells were seeded in a 96-well plate at a density of 10^4^ cells per well and cultured for 24 h before adding different ferroptosis inhibitors, including ferrostain-1 (Fer-1, 100 nmol/L), deferoxamine (DFO, 100 μmol/L), and vitamin E (VE, 20 μmol/L). Subsequently, the cells were subjected to 8.5 W HIFU irradiation for 10 s, 30 s, 50 s, and 70 s, respectively, followed by a 24-h incubation. Cell viability was assessed using a 10% CCK-8 solution according to the routine protocol. To validate the ferroptosis of other cells treated with HIFU, both CT26 cells and TC1 cells were treated with 8.5 W HIFU for 50 s in the presence of different ferroptosis inhibitors and cell viability was measured by CCK-8 as described above.

#### 2.3.4 Live/dead cell staining of 4T1 cells after HIFU treatment

4T1 cells were seeded in a 12-well plate and cultured for 24 h. Subsequently, the cells underwent HIFU irradiation (8.5 W) were irradiated for 10, 30, 50, and 70 s, respectively, and were further cultured for 24 h 4T1 cells after various treatments as described above were stained with Calcein-AM and PI dispersed in PBS for 15 min. After washing with PBS three times, cells were analyzed using a flow cytometer (BD Biosciences, East Rutherford, USA).

#### 2.3.5 Effect of HIFU on GSSG *in vitro*

GSSG was diluted to 500 μmol/L with water, irradiated with 8.5W HIFU for 5 min, and subsequently filtered through a 0.45 μm nylon filter. Untreated GSSG served as control. The samples were analyzed with high-performance liquid chromatography (HPLC, Shimadzu, Japan) using an isocratic solvent system (10% acetonitrile and 90% water containing 0.05% trifluoroacetic acid) and detection at 214 nm. In addition, the GSSG concentration before and after HIFU treatment was also quantified using a GSH/GSSG detection kit.

#### 2.3.6 *In vitro* GSH/GSSG levels in tumor cells treated with HIFU in the presence of ferroptosis inhibitors

4T1 cells were seeded onto 12-well chamber slides and cultured overnight, and then 4T1 cells were added with various ferroptosis inhibitors, including ferrostain-1 (Fer-1, 100 nmol/L), deferoxamine (DFO, 100 μmol/L), and vitamin E (VE, 20 μmol/L) before various treatments. Subsequently, the cells underwent 8.5 W HIFU irradiation and were irradiated for 0, 10, 30, 50, and 70 s, respectively and were further cultured for 1 h. Cells from the different treatment groups were collected, lysed and centrifuged, and the supernatant was used for the GSH/GSSG detection kit. To determine GSH/GSSG levels in other cells, both CT26 cells and TC1 cells were treated with 8.5 W HIFU for 50 s in the presence of various ferroptosis inhibitors and analyzed using a GSH/GSSG detection kit as described above.

#### 2.3.7 *In vitro* ROS level in tumor cells treated with HIFU in the presence of ferroptosis inhibitors

4T1 cells were seeded onto 12-well chamber slides and cultured overnight, and then 4T1 cells were added with various ferroptosis inhibitors, including ferrostain-1 (Fer-1100 nmol/L), deferoxamine (DFO, 100 μmol/L), and vitamin E (VE, 20 μmol/L) before various treatments. Subsequently, the cells underwent 8.5 W HIFU irradiation and were irradiated for 0, 10, 30, 50, and 70 s, respectively and were further cultured for 1 h. The cells were incubated with DCFH-DA (1:2000) for 20 min, and washed twice with serum-free medium. The fluorescence signal of ROS in the cells was then analyzed with a FACS Calibur flow cytometer (BD Biosciences, East Rutherford, USA). To determine the ROS level in other cells, both CT26 and TC1 cells were treated with 8.5 W HIFU for 50 s in the presence of various ferroptosis inhibitors, stained with DCFH-DA, and then the intracellular ROS content was measured with flow cytometry as described above.

### 2.4 Validation of NP-PF to enhance HIFU-induced ferroptosis in 4T1 cells

#### 2.4.1 Fabrication and characterization of nanoenhancers

PLGA nanoparticles loaded with PFOB (NP-PF) as nanoenhancers of HIFU were prepared using a nanoprecipitation method. Specifically, a solution of PLGA-OH (5 mg), PFOB (0.96 mg), and DSPE-PEG2000 (0.5 mg) in 0.5 mL of acetone was prepared as the organic phase. Afterward, the organic solution was rapidly injected into 1 mL of water. Subsequently, acetone was removed by vacuum evaporation, resulting in the NP-PF suspension. PLGA nanoparticles without PFOB (NP) were prepared with the same method as NP-PF except that the organic phase did not include PFOB.

The hydrated particle size and zeta potential of NP and NP-PF were measured using the dynamic light scattering method (Malvern, England). The drug loading of PFOB was determined by dissolving NP-PF in CDCl_3_ containing perfluoro-15-crown-5-ether as an internal standard and measuring it using ^19^F-NMR^31^. The *in vitro* stability of NP-PF was evaluated by monitoring the changes in particle size and zeta potential of NP-PF at 4 °C in PBS solution over a week.

#### 2.4.2 Cell viability and cell apoptosis of 4T1 cells treated with NP-PF + H

To investigate the enhanced therapeutic effects of NP-PF on HIFU, 4T1 cells were seeded in a 96-well plate at a density of 10^4^ cells per well and cultured for 24 h. Cells from the HIFU, NP + H, and NP-PF + H groups were respectively incubated with PBS, NP, and NP-PF for 12 h, followed by HIFU irradiation (8.5 W, 30 s). Subsequently, cells were further incubated for 12 h. Afterward, the CCK-8 assay was performed to assess cell viability.

The assessment of apoptosis in 4T1 cells treated with NP-PF + H was conducted using the Annexin V-FITC/PI Cell Apoptosis Kit following the manufacturer's guidelines. 4T1 cells were seeded onto 12-well chamber slides and cultured overnight. Then 4T1 cells were first incubated with NP or NP-PF for 8 h, followed by treatment with 8.5 W HIFU for 30 s. After 24 h of incubation, apoptosis of the cells was assessed as described above. Cells treated with NP-PF or HIFU only were used as controls.

#### 2.4.3 Cell viability of 4T1 cells treated with NP-PF + H in the presence of ferroptosis inhibitors

To validate that NP-PF enhances the ferroptosis of 4T1 cells treated with HIFU, cell viability of 4T1 cells was tested after NP-PF + H treatment in the presence of ferroptosis inhibitors. Briefly, 4T1 cells were seeded in a 96-well plate at a density of 10^4^ cells per well and cultured for 24 h before adding different ferroptosis inhibitors, including ferrostain-1 (Fer-1, 100 nmol/L), deferoxamine (DFO, 100 μmol/L), and vitamin E (VE, 20 μmol/L). Subsequently, cells were subjected to 8.5 W HIFU irradiation for 10, 30, 50, and 70 s, respectively, followed by a 24-h incubation. Cell viability was assessed using a 10% CCK-8 solution according to the routine protocol.

#### 2.4.4 LPO level in 4T1 cells treated with NP-PF + H

4T1 cells were seeded onto 12-well chamber slides and cultured overnight. Subsequently, 4T1 cells were first incubated with NP or NP-PF for 8 h, followed by treatment with 8.5 W HIFU for 30 s. After 1-h incubation, cells were incubated with C11-BODIPY at 37 °C for 30 min, followed by three washes with PBS. The fluorescence signal of LPO in the cells was analyzed with a FACS Calibur flow cytometer (BD Biosciences, East Rutherford, USA).

#### 2.4.5 ROS and GSH/GSSG levels in 4T1 cells treated with NP-PF + H

4T1 cells were seeded onto 12-well chamber slides and cultured overnight. Subsequently, 4T1 cells were first incubated with NP or NP-PF for 8 h, followed by treatment with 8.5 W HIFU for 30 s. After 1 h of incubation, cells were incubated with DCFH-DA (1:2000) for 20 min, and washed twice with serum-free medium. The fluorescence signal of ROS in the cells was then analyzed with a FACS Calibur flow cytometer (BD Biosciences, East Rutherford, USA). For the determination of intracellular GSH and GSSG content, after 1 h of incubation, cells from the different treatment groups were collected, lysed and centrifuged, and the supernatant was used for GSH/GSSG detection kit. Cells treated with NP-PF or HIFU only were used as controls.

#### 2.4.6 Western blotting analysis of ferroptosis-related proteins in 4T1 cells treated with NP-PF + H

4T1 cells were seeded into 6-well plates and incubated with PBS, NP or NP-PF overnight. After 8 h, the HIFU group was irradiated with 8.5 W–HIFU for 30 s, followed by 12 h incubation to collect cells. Proteins were extracted using the Lysis and Extraction Buffer. After boiling the protein samples for 10 min, equal amounts of each sample were loaded onto 10% SDS-polyacrylamide gels for electrophoretic separation and subsequently transferred to PVDF membranes. Following a 1-h blocking step, the membranes were incubated overnight at 4 °C with primary antibodies (anti-SCL7A11 and anti-GPX4), followed by a 2-h incubation with secondary antibodies. An anti-GAPDH antibody served as a loading control. The protein bands were visualized using X-ray film and subjected to densitometry analysis using the Gel-Pro Analyzer software.

#### 2.4.7 mRNA expressions in 4T1 cells treated with NP-PF + H

4T1 cells were initially seeded in a 6-well plate, cultured overnight, and then subjected to treatment with either NP-PF or PBS for a duration of 8 h. Following this, the HIFU treatment groups were exposed to HIFU at a power of 8.5 W for 30 s and then further incubated for an additional 12 h. Post-treatment, the cells were harvested for RNA isolation, which was performed using Trizol reagent in accordance with the supplier's guidelines. The extracted mRNA was purified through a two-step process employing Dynabeads Oligo (dT) (Thermo Fisher Scientific, CA, USA), followed by reverse transcription into complementary DNA (cDNA). This cDNA served as a template for the synthesis of second-strand DNAs labeled with uracil (U). The ligated DNA products were subsequently treated with the heat-labile uracil DNA glycosylase (UDG) enzyme (NEB, cat. m0280, USA) to remove uracil, and then amplified *via* PCR. The amplified DNA fragments were sequenced using a 2 × 150bp paired-end (PE150) approach on an Illumina NovaseqTM 6000 platform (LC-Bio Technology Co., Ltd., Hangzhou, China), adhering to the manufacturer's recommended protocol. The resulting data were analyzed to identify differentially expressed genes that were significantly enriched in Gene Ontology (GO) terms and Kyoto Encyclopedia of Genes and Genomes (KEGG) metabolic pathways, employing GO functional enrichment and KEGG analysis methodologies.

### 2.5 DC maturation induced by NP-PF + H-treated tumor cells

Bone marrow-derived dendritic cells (BMDCs) were isolated from female BALB/c mice following an established protocol^34^. The cells were then cultured in RPMI-1640 cell culture medium supplemented with GM-CSF (20 ng/mL) and IL-4 (10 ng/mL). After 5 days of culture, BMDCs were suspended in fresh culture medium in the receptor well of 12-well Transwell® plates and cocultured with 4T1 cells treated with PBS, NP-PF, HIFU, NP + H, and NP-PF + H, respectively, which were seeded in the donor well. Following a 24-h coculture, BMDCs were stained successively with anti-CD11c-FITC, anti-CD80-PE, and anti-CD86-APC for DC maturation analysis using flow cytometry (BD Biosciences, East Rutherford, USA). The concentrations of TNF-*α* and IFN-*γ* in the supernatant of the receptor wells were quantified using ELISA kits (Multisciences, China).

To verify that BMDC maturation was triggered by ferroptosis of tumor cells, 4T1 cells were added with various ferroptosis inhibitors, including ferrostain-1 (Fer-1, 100 nmol/L), deferoxamine (DFO, 100 μmol/L), and vitamin E (VE, 20 μmol/L) before various treatments and then were incubated with BMDC. BMDC maturation and cytokine secretion were detected as described above.

### 2.6 Anti-tumor efficacy of NP-PF + H treatment

#### 2.6.1 Tumor growth inhibition after NP-PF + H treatment

The 4T1 tumor-bearing mice were randomly divided into 5 groups, with 12 mice in each group. The tumor volume was monitored from the beginning of tumor seeding, and UTGR was calculated according to the following Eq. (3) and the curve was drawn^35^.

(3) $$\text{UTGR}=\left\{\left[V\left(t_{n+2}\right)-V\left(t_{n}\right)\right]/V\left(t_{n}\right)\times 100\%\right\}‒\left\{\left[V\left(t_{n}\right)‒V\left(t_{n-2}\right)\right]/V\left(t_{n-2}\right)\times 100\%\right\}$$

where *V(t*_*n*_*)* is the mean tumor volume on Day *n* and *t* means time, in days.

When the tumor grew to UTGR = 0 (Day 5), 6 mice in each group were selected to receive different treatments. The mice in the PBS group and NP-PF group were intravenously injected with PBS and NP-PF (0.75 mg), respectively. The mice in HIFU-related groups were injected with the same amount of NP or NP-PF. After 3 h, the tumors were irradiated with 8.5 W HIFU for 30 s. The tumor growth and body weight of mice in each group were monitored, and the tumors were dissected and weighed, and photographed on Day 13. When the remaining mice grew to UTGR >0 (Day 7), the same treatment was performed and tumor growth was subsequently monitored.

#### 2.6.2 HE, TUNEL, and LPO staining of tumor slices after NP-PF + H treatment

The 4T1 tumor-bearing mice were randomly divided into 5 groups, 9 mice in each group. When the tumor grew to UTGR = 0 (Day 5), the mice in the PBS group and NP-PF group were intravenously injected with PBS and NP-PF (0.75 mg), respectively. The mice in HIFU-related groups were injected with the same amount of NP or NP-PF and received HIFU treatments (8.5 W 30 s) after 3 h. After 1 day of treatment, 6 mice in each group were sacrificed, and the tumor sites were dissected and embedded in sections for hematoxylin and eosin (H&E) staining and TdT-mediated dUTP Nick-End Labeling (TUNEL) staining. Three days later, the remaining mice were harvested for tumor dissection, and cryosections were prepared for immunofluorescence staining of LPO with C11-BODIPY. The stained slices were scanned using Pannoramic P-MIDI (3DHISTECH Ltd., Magyarország).

#### 2.6.3 Assessment of model mice survival after NP-PF + H treatment

The 4T1 tumor-bearing mice were randomly divided into 5 groups, 6 mice in each group. When the tumor grew to UTGR = 0 (Day 5), the mice in the PBS group and NP-PF group were intravenously injected with PBS and NP-PF (0.75 mg), respectively. The mice in HIFU-related groups were injected with the same amount of NP or NP-PF and received HIFU treatments (8.5 W 30 s) after 3 h. The survival of the mice was subsequently monitored and recorded. The death of the mice was defined as the tumor volume exceeding 1500 mm^3^.

### 2.7 Evaluation of anti-tumor immune responses in model mice after NP-PF + H treatment

The 4T1 tumor-bearing mice were randomly divided into 5 groups, 5 mice in each group. When the tumor grew to UTGR = 0 (Day 5), the mice in the PBS group and NP-PF group were intravenously injected with PBS and NP-PF (0.75 mg), respectively. The mice in HIFU-related groups were injected with the same amount of NP or NP-PF and received HIFU treatments (8.5 W 30 s) after 3 h. Tumors, tumor-draining lymph nodes, and spleens were harvested on Day 7 after different treatments and prepared as single-cell suspensions, respectively. Lymphocyte and tumor cell suspensions were analyzed for DC maturation by staining with anti-CD11c-FITC, anti-CD80-PE, and anti-CD86-PE/Cy7 and for T cell proportion by staining with anti-CD3-AF700, anti-CD4-FITC, and anti-CD8-PE. Immune-suppressive regular T cells (Treg cells) in tumor cell suspensions were stained with anti-CD45-BV510, anti-CD3-AF700, anti-CD4-FITC, and anti-CD25-APC. Afterward, Treg cells were further stained with anti-FOXP3-PE after membrane rupture. MDSC cells were stained with anti-CD45-BV510, anti-CD11b-PE/Cy7, anti-Gr-1-APC, and M1/2 type tumor-associated macrophages in tumors were stained with anti-CD45-APC, anti-F4/80-AF488, anti-CD86-PE, and anti-CD206-PerCP-Cy5.5. Anti-CD45-BV510, anti-CD3-AF700, anti-CD8-FITC, anti-CD44-PE, and anti-CD62L-APC staining for single-cell suspensions from the spleen was used to assess the proportion of central memory cells. After staining, the cells were bathed in ice for 20 min, washed with PBS, and detected by flow cytometry (BD Biosciences, East Rutherford, USA).

### 2.8 *In vivo* safety evaluation of NP-PF

Healthy BALB/c mice were divided into two groups and injected intravenously with PBS or NP-PF, respectively. After 7 days, the major organs including the heart, liver, spleen, lung, and kidneys were dissected, fixed in paraformaldehyde, and sectioned for H&E staining. At the same time, blood samples were collected for blood routine and blood biochemical tests. Blood routine indexes included the ratio of lymph, the concentrations of red blood cells (RBCs), white blood cells (WBCs), hemoglobin (HGB), platelets (PLTs), mean corpuscular volume (MCV), mean platelet volume (MPV), red cell volume distribution width (RDW), mean corpuscular hemoglobin (MCH), platelet distribution width (PDW), and blood biochemical indexes included aspartate aminotransferase (AST), alkaline phosphatase (ALP), alanine transaminase (ALT), creatinine (CRE), and blood urea nitrogen (BUN).

### 2.9 Statistical analysis

All data were plotted and analyzed using GraphPad Prism 8.0, and all data are expressed as mean ± standard deviation (SD). The Student's *t*-test was used to analyze the data between two groups, and the One-way analysis of variance (ANOVA) followed by Tukey's *post hoc* test was used to analyze the data between multiple groups.

## 3 Results

### 3.1 HIFU-induced ferroptosis across diverse energy efficiency factors

**Figure 1:**
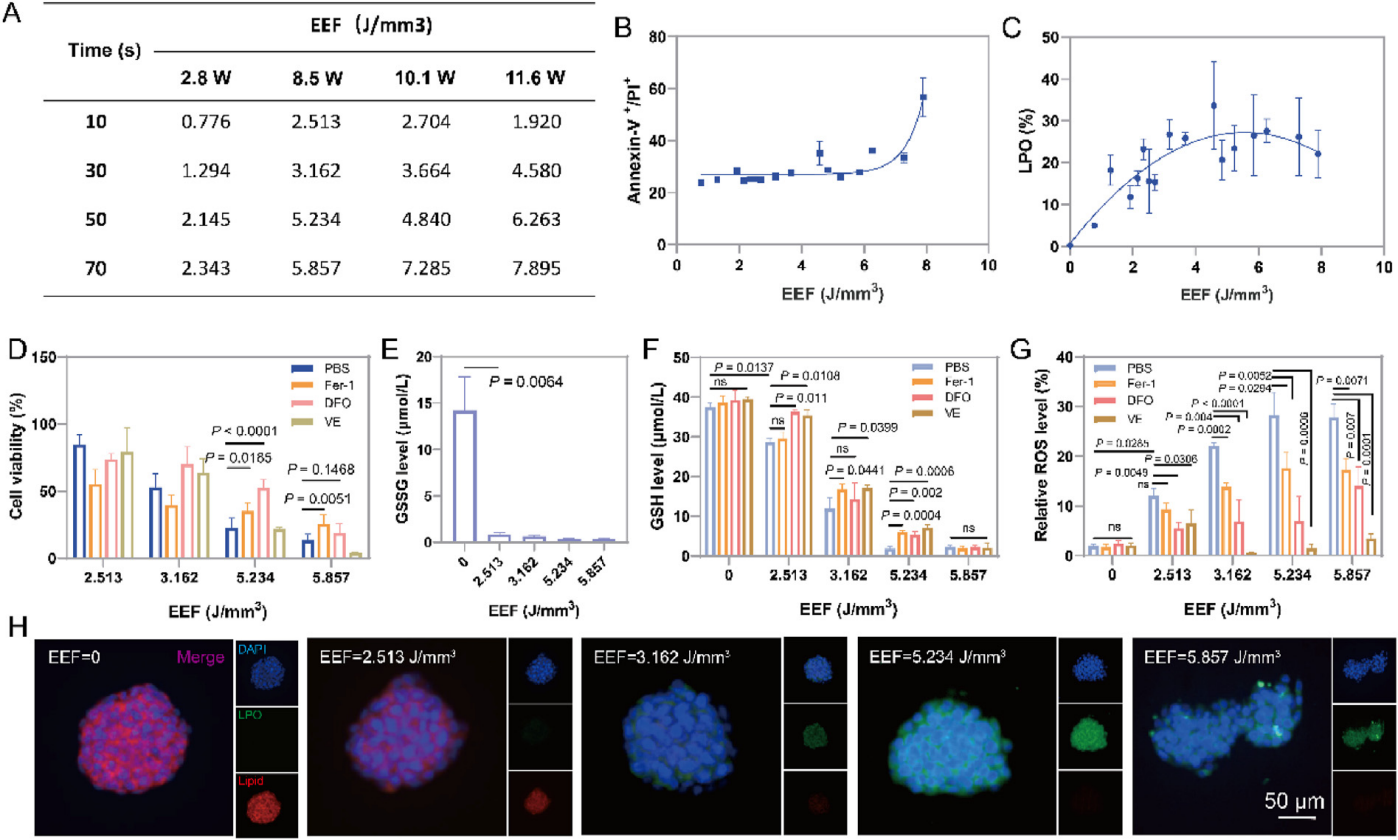
HIFU-induced ferroptosis across diverse energy efficiency factors (EEF). (A) Corresponding EEF values of HIFU treatments with different powers and times. (B, C) The apoptosis rate (B) and lipid peroxidation level (C) of 4T1 cells treated with HIFU at different EEF values (*n* = 3). (D) The cell viability of 4T1 cells treated with HIFU at different EEF values (*n* = 3). (E) The GSSG content in 4T1 cells treated with HIFU at different EEF values (*n* = 3). (F, G) The GSH content (F) and ROS content (G) in 4T1 cells treated with HIFU at different EEF values (*n* = 3). (H) Fluorescence imaging of 4T1 cells treated with HIFU at different EEF values after staining with BODIPY-C11, showing the intracellular distribution of LPO (green fluorescence) and normal lipids (red fluorescence). Data are expressed as mean ± SEM. Scale bar = 50 μm.

Different energy levels of HIFU can induce varying degrees of ablative capacity in tumor tissues. To quantitatively assess the energy efficiency of HIFU, we employed the energy efficiency factor (EEF), which represents the ultrasound energy required to ablate 1 mm^3^ of tissue, as a factor for evaluating energy efficiency^36^. Initially, we utilized porcine liver tissue to explore the ablative capabilities of HIFU at different power levels and durations. It was revealed that as the HIFU power and duration increased, the lateral damage zone in the porcine liver expanded (Supporting Information Fig. S1A‒S1C). However, there was no significant difference in the ablation depth, emphasizing the importance of enhancing HIFU penetrability. The corresponding EEF for each parameter was calculated based on the ablation volume in the porcine liver (Fig. 1A). Next, we investigated the apoptotic and ferroptotic effects of HIFU at different energy levels on 4T1 cells. As shown in Supporting Information Fig. S2A, the proportion of apoptotic cells increased with treatment durations at four different power levels. Lower-power HIFU (2.8, 8.5, and 10.1 W) maintained relatively low levels of apoptosis in tumor cells. However, when the power was increased to 11.6 W, the apoptotic effect on 4T1 cells doubled. At 2.8 W, HIFU induced a relatively low level of LPO in tumor cells, while higher-power HIFU induced an LPO peak at 30 s, after which the proportion of LPO-positive cells either maintained at this level (8.5 and 10.1 W) or gradually declined (11.6 W) (Fig. S2B). When the EEF of HIFU was fitted with the apoptotic and LPO levels (Fig. 1B and C), it was found that HIFU with an EEF value lower than 7.285 J/mm^3^ had similar effects on the apoptosis of 4T1 cells while a higher EEF value (7.895 J/mm^3^) generated a stronger apoptotic level on 4T1 cells. Interestingly, under a certain EEF, LPO production in 4T1 cells increased with EEF and peaked when the EEF was 5.234 J/mm^3^. When the EEF was above 5.234 J/mm^3^, LPO production in 4T1 cells even decreased with EEF. These results suggest that certain EEF is required for LPO generation in tumor cells.

We then selected a set of HIFU powers to further assess their ferroptotic effects on 4T1 cells. Combining cytotoxicity assays and cell death staining, it was evident that higher HIFU EEF resulted in increased cell cytotoxicity and cell death (Fig. 1D and Supporting Information Fig. S3). Pre-treatment with a ferroptosis inhibitor (Fer-1 or DFO) significantly alleviated the cytotoxicity induced by HIFU when the EEF value was above 3.162 J/mm^3^, indicating the occurrence of cell ferroptosis after HIFU treatment (Fig. 1D). Glutathione (GSH) and its oxidized form glutathione disulfide (GSSG) are key molecules in the maintenance of redox balance in tumor cells^37^. GSSG is formed by two GSH molecules through a disulfide bond between cysteine residues^38^. According to the mechanochemical principle, the shear stress generated by HIFU may break the disulfide bond in the GSSG structure and destroy its activity^20^. It was found that the GSSG content in 4T1 cells decreased sharply as expected under the action of HIFU with various EEF values (Fig. 1E). Further, HIFU was performed directly on GSSG *in vitro*. The results of HPLC and quantitative analysis showed that HIFU treatment reduced the concentration of GSSG from 500 to 60.95 ± 0.51 μmol/L (Supporting Information Fig. S4). Accordingly, HIFU treatment with various EEF values significantly decreased the GSH level (Fig. 1F) and significantly increased the ROS level (Fig. 1G) in 4T1 cells. Importantly, both GSH consumption and ROS production peaked when the EEF value of HIFU reached 5.234 J/mm^3^. Interestingly, the addition of ferroptosis inhibitors significantly suppressed ROS production induced by HIFU treatment with various EEF values (Fig. 1G). Moreover, the addition of ferroptosis inhibitors significantly alleviated the glutathione depletion induced by HIFU treatment (except EEF = 5.857 J/mm^3^) (Fig. 1F). The results obtained from confocal imaging analysis of LPO following varying durations of HIFU treatment were in agreement with the findings observed through flow cytometry. These results demonstrated that the fluorescence intensity of LPO was notably elevated after HIFU irradiation, particularly when the EEF value reached 5.234 J/mm^3^ (Fig. 1H). All these results suggest that within a certain range, the higher EEF of HIFU, the stronger the ferroptosis effect is triggered by the imbalance of the cellular redox system. In addition, we also performed ferroptosis validation in CT26 cell and TC1 cell lines. The killing effect of HIFU on both cell types gradually enhanced with increasing EEF (Supporting Information Figs. S5 and S6). HIFU treatment with the EEF value of 5.234 J/mm^3^ could drastically reduce the GSSG and GSH contents in CT26 and TC-1 cells, thereby undermining the GSH/GSSG balance and causing a surge of intracellular ROS and LPO generation (Figs. S5 and S6). The presence of ferroptosis inhibitors could also alleviate the killing effect of HIFU on tumor cells to a certain extent (Figs. S5F and S6F). These results indicated that appropriate HIFU parameters could also trigger ferroptosis in CT26 and CT-1 cells. In conclusion, the induction of ferroptosis in 4T1 cells by HIFU treatment has its limitations. To enhance the ferroptosis effect, it is essential to carefully select and optimize the therapeutic parameters.

### 3.2 Nano-enhancers augmented the ferroptosis of tumor cells induced by HIFU

**Figure 2:**
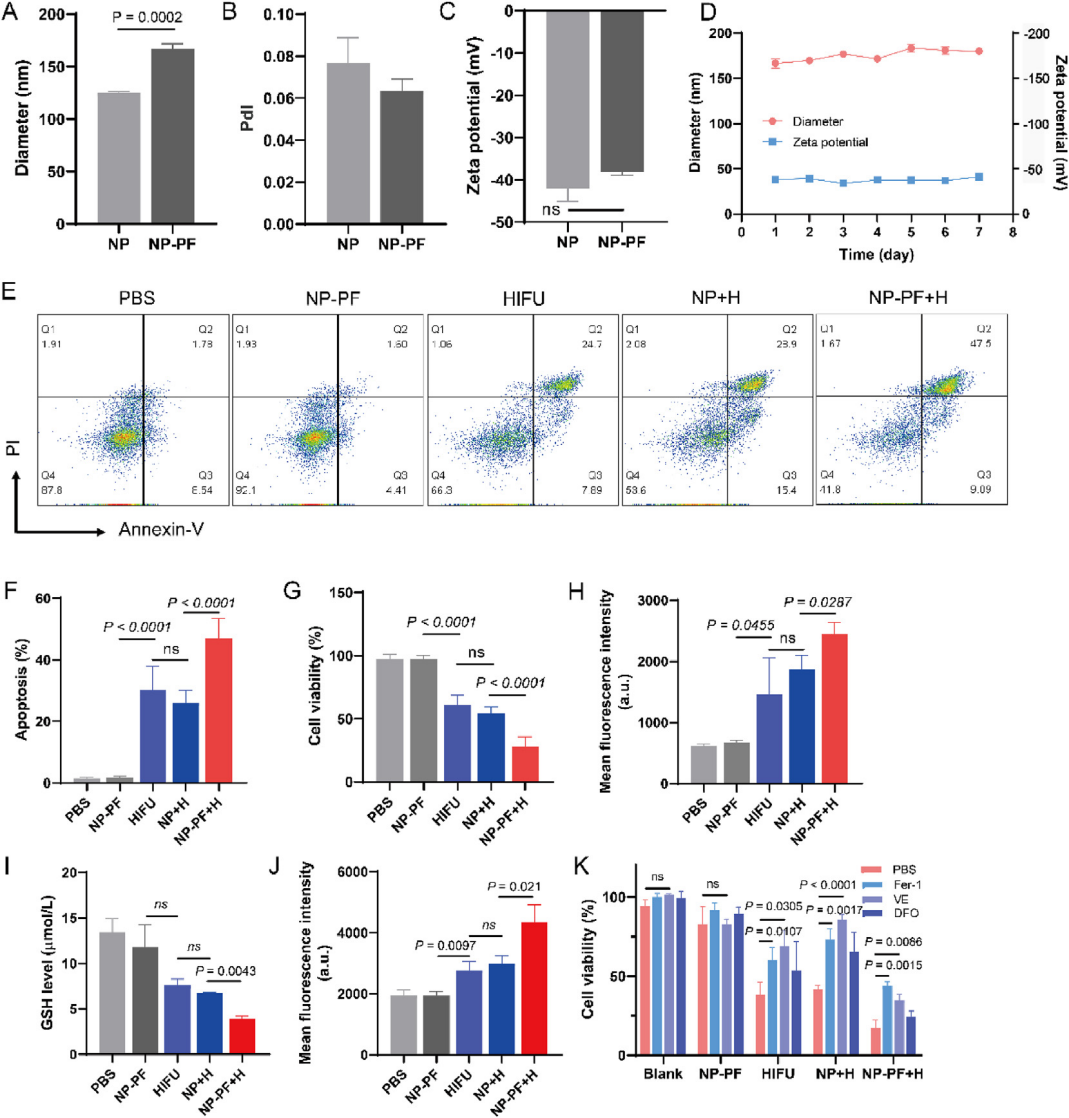
NP-PF enhanced HIFU-induced ferroptosis in 4T1 cells. (A–C) The particle size (A), polydisperse index (PdI) (B), and zeta potential (C) of NP and NP-PF (*n* = 3). (D) The particle size and zeta potential stability of NP-PF over 7 days (*n* = 3). (E, F) Representative flow cytometry plots (E) and the corresponding quantitative results (F) of tumor cell apoptosis after different treatments (*n* = 3). (G) The cell viability of 4T1 cells after different treatments obtained by the CCK8 assay (*n* = 6). (H–J) Intracellular ROS level (H), intracellular GSH content, (I) and lipid peroxidation (LPO) level (J) in 4T1 cells after different treatments (*n* = 3). (K) The cell viability of 4T1 cells that received various treatments in the presence of different ferroptosis inhibitors including Fer-1, DFO, and VE (*n* = 3). Data are expressed as mean ± SEM.

PFOB was encapsulated into PEG-modified PLGA nanoparticles using a nano-precipitation method, yielding the nano-enhancer-designated NP-PF. The average hydrodynamic diameter of NP-PF, measured by dynamic light scattering was 166.5 nm, indicating a 42 nm increase compared to bare PLGA nanoparticles (NP) (Fig. 2A). Moreover, the size distribution of NP-PF was uniform, with a low polydispersity index (PDI) of approximately 0.06 (Fig. 2B). There was no significant difference in the zeta potential between NP and NP-PF, with values measuring around −42 and −38 mV, respectively (Fig. 2C). The prepared nano-enhancer demonstrated excellent size and potential stability over the course of 7 days (Fig. 2D). The drug loading capability (DLC) and the encapsulation efficiency (EE) of PFOB in NP-G/P were 18.24 ± 1.50% and 95.00 ± 1.07%, respectively. In determining the optimal HIFU treatment power, we conducted a balanced analysis of the relevant outcomes in Fig. 1. We selected the EEF value of 3.162 J/mm^3^ as the treatment parameter for subsequent experiments, which could elicit a high level of LPO in 4T1 cells while minimizing possible damage to normal surrounding tissues, in contrast to higher energy settings. To evaluate the cytotoxicity of the nano-enhancer, we initially conducted a CCK-8 assay on 4T1 cells. NP-PF itself demonstrated no toxicity towards tumor cells, while both HIFU and NP + H groups resulted in approximately 50% viability. Notably, NP-PF significantly augmented HIFU-induced cytotoxicity, reaching 27.7% viability against 4T1 cells (Fig. 2E‒G). Apoptosis detection in 4T1 cells further supported the enhanced effect of NP-PF on HIFU-induced tumor cell destruction, exhibiting a higher apoptosis rate (46.9%) compared to HIFU treatment (30.1%) or NP + H treatment (30.1%) (Fig. 2E‒G). To elucidate the role of NP-PF in HIFU-triggered ferroptosis in tumor cells, we assessed relevant ferroptosis indicators in 4T cells following different treatments. In comparison to the PBS group, HIFU induction resulted in increased ROS generation, increased intracellular GSSG and GSH depletion, and enhanced LPO production (Fig. 2H‒J and Supporting Information Fig. S7). The introduction of the nano-enhancer (NP-PF) significantly elevated intracellular ROS levels, further depleting intracellular GSSG and GSH, and achieving the highest degree of LPO in tumor cell membranes. Conversely, the NP + H group exhibited no significant differences in ROS, GSH, and LPO levels compared to the HIFU group. To rigorously confirm the ferroptotic nature of cell death triggered by NP-PF + H treatment, various ferroptosis inhibitors, including DFO, Fer-1, and Vitamin E, were pre-administered on 4T1 cells before various treatments. As illustrated in Fig. 2K, Fer-1 and Vitamin E demonstrated a significant attenuation of cytotoxicity in the HIFU, NP + H, and NP-PF-H groups, providing further evidence of the ferroptosis occurrence.

### 3.3 Validation of ferroptosis triggered by NP-PF + H treatment

**Figure 3:**
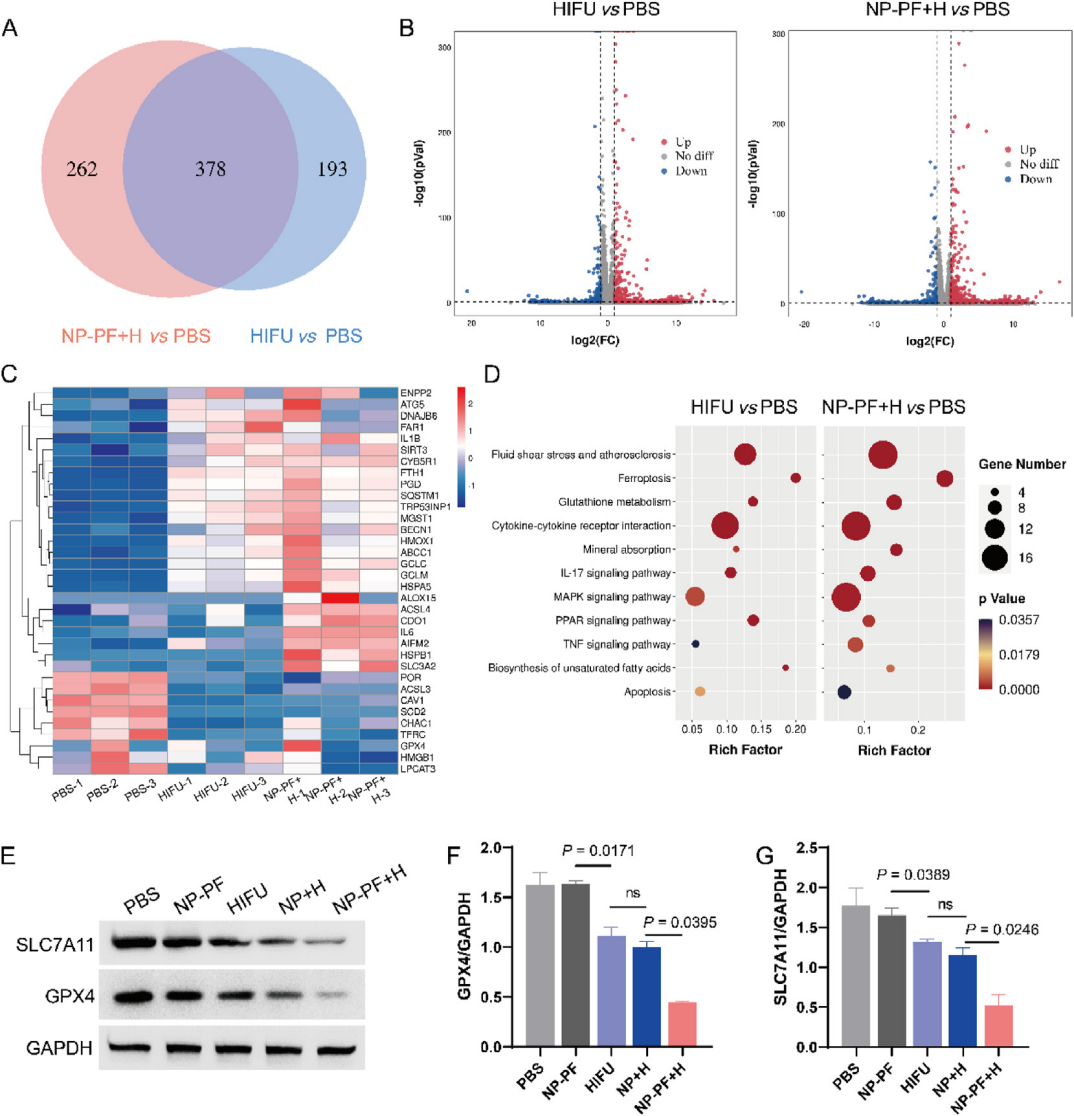
Validation of ferroptosis triggered by NP-PF + H treatment. (A) Venn diagram of gene expressions in 4T1 cells after HIFU or NP-PF + H treatment when compared to PBS treatment. (B) Volcano plot of gene expressions in 4T1 cells after treatment with HIFU or NP-PF + H compared with PBS. The screening criteria were FDR<0.05 and |log2fold change|=>1. (C) Heat map of differential gene expression in 4T1 cells after different treatments (*n* = 3). The number in the scale plate referred to the log2fold change. (D) The pathways in which differentially expressed genes in 4T1 cells treated with HIFU or NP-PF + H enriched by the KEGG enrichment analysis. (E–G) Representative Western blotting analysis (E) and the corresponding quantitative results of GPX4 (F) and SLC7A11 (G) proteins in 4T1 cells after different treatments (*n* = 3). Data are expressed as mean ± SEM.

To further confirm the occurrence of HIFU-induced ferroptosis in tumor cells, we conducted RNA sequencing analysis on 4T1 cells treated with HIFU and NP-PF + H. The results of Venn diagram showed that there were 378 identical differentially expressed genes and 262 unique differentially expressed genes in the NP-PF + H *versus* PBS group compared with the HIFU *versus* PBS group, which implied the difference between the three treatment groups (Fig. 3A). These differentially expressed genes between treatment groups were presented in a volcano plot (Fig. 3B). As shown in Fig. 3C, compared to PBS treatment, HIFU treatment significantly upregulated the expressions of ferroptosis-driver genes such as cytochrome *b*5 reductase 1 (*CYB5R1*), fatty acyl-CoA reductase 1 (*FAR1*), interleukin 1 beta (*IL1B*), FYVE, RhoGEF And PH Domain (*FGD*), sirtuin 3 (*SIRT3*), and concurrently downregulated the expression of ferroptosis-suppressor genes such as glutathione peroxidase 4 (*GPX4*), caveolin 1 (*CAV1*), stearoyl-CoA desaturase (*SCD*), long-chain acyl-CoA synthetase 3 (*ACSL3*), thereby promoting the occurrence of ferroptosis. Upon the addition of the nano-enhancer, NP-PF significantly enhanced HIFU-induced ferroptosis in tumor cells, leading to a significant increase in the number and expression levels of ferroptosis-related genes (Fig. 3B and C). Particularly, the expression of ferroptosis-related genes like long-chain acyl-CoA synthetase 4 (*ACSL4*), cytosolic dopamine beta-hydroxylase (*CDO1*), interleukin 6 (*IL6*) significantly increased in the NP-PF + H group, whereas they showed no significant changes in the HIFU group compared with the PBS group. Pathway enrichment analysis revealed that HIFU treatment significantly upregulated pathways associated with fluid shear stress and atherosclerosis, ferroptosis, glutathione metabolism, and mineral absorption in tumor cells (Fig. 3D). The inclusion of NP-PF not only amplified these enrichment factors but also augmented the number of genes involved in these pathways enriched in these categories. The decrease of GSH and GSSG contents could inhibit the activity of GPX4 and disable its anti-peroxidation function^39^. The heat map presented a series of differentially expressed redox-related genes in the HIFU or NP-PF + H group, compared to the PBS group (Supporting Information Fig. S8), which illustrated that redox processes occurred in tumor cells after HIFU or NP-PF + H treatment. In addition, the disruption of the intracellular antioxidant system also leads to the down-regulation of solute carrier family 7 member 11 (*SLC7A11*), a transmembrane protein that mediates cysteine/glutamate transport activity in the xc^‒^ system, which contributes to cell ferroptosis^40^. We confirmed the expression of these pertinent ferroptosis proteins (GPX4 and SLC7A11) *via* Western blot (WB) experiments. NP-PF exhibited no impact on the expression of these proteins (Fig. 3E‒G). Conversely, the HIFU and NP + H groups demonstrated a significant downregulation in the expressions of GPX4 and SLC7A11 proteins, and NP-PF + H treatment more markedly inhibited these proteins which will be beneficial to ferroptosis. These findings further support the occurrence of ferroptosis in 4T1 cells following NP-PF + H treatment.

### 3.4 Nano-enhancer potentiated dendritic cell maturation triggered by HIFU-treated tumor cells

**Figure 4:**
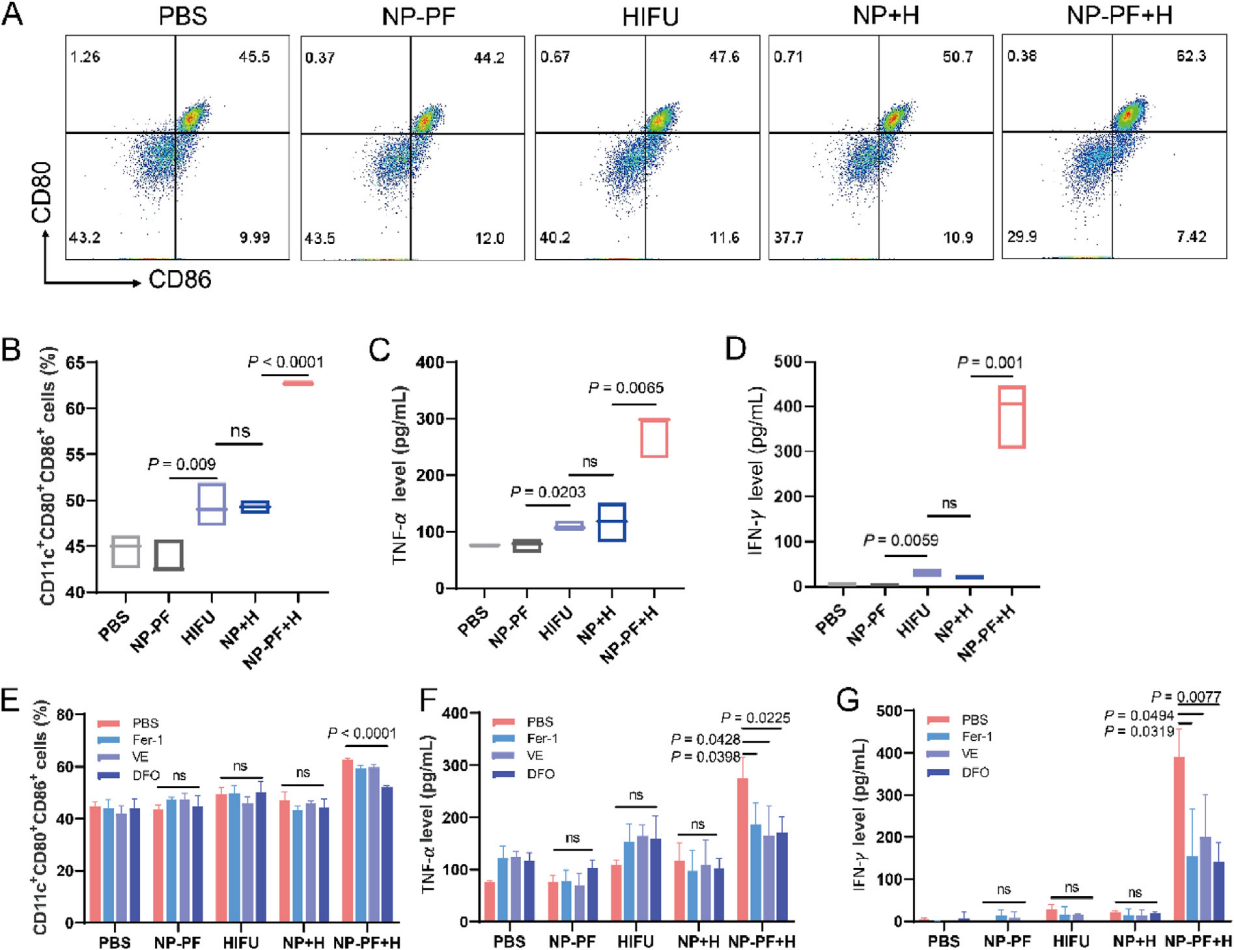
NP-PF potentiated dendritic cell maturation triggered by HIFU-induced cell ferroptosis. (A–B) Representative flow cytometry plots (A) and the corresponding quantitative results (B) demonstrated the maturation of BMDCs triggered by 4T1 cells from different treatment groups (*n* = 3). (C, D) TNF-*α* (C) and IFN-*γ* (D) concentrations in the cell culture medium of BMDCs co-incubated with 4T1 cells from different treatment groups (*n* = 3). (E) The maturation of BMDCs triggered by 4T1 cells pretreated with different formulations in the presence of various ferroptosis inhibitors (*n* = 3). (F, G) TNF-*α* (F) and IFN-*γ* (G) concentrations in the cell culture medium of by BMDCs co-incubated with 4T1 cells pretreated with different formulations in the presence of different ferroptosis inhibitors and achieved different treatments (*n* = 3). Data are expressed as mean ± SEM.

After HIFU treatment, it was found that immune-related pathways within 4T1 cells, including the IL17 signaling pathway, MAPK signaling pathway, PPAR signaling pathway, and TNF signaling pathway, exhibit higher enrichment scores through KEGG enrichment analysis (Fig. 3B). The NP + H group also demonstrated varying degrees of enhancement in these pathways. Following the co-cultivation of HIFU-treated 4T1 cells with bone marrow-derived dendritic cells (BMDC), we assessed the maturation of stimulated BMDC cells. It was demonstrated that the proportions of CD80^+^CD86^+^ cells in the HIFU and NP+H groups were slightly higher than the PBS group, only from 44.6% to 49.4% and 49.3%, respectively. However, after NP-PF + H treatment, 4T1 cells triggered the maturation of 62.7% of BMDC cells (Fig. 4A and B). Concurrently, the secretion of relevant cytokines including TNF-*α* and INF-*γ* in the culture supernatant also significantly increased in the NP-PF + H group (Fig. 4C and D). In contrast, other treatment groups did not exhibit superior cytokine secretion. To investigate whether the BMDC maturation was induced by tumor cell ferroptosis, we introduced various ferroptosis inhibitors and observed changes in the BMDC cell maturation. The results revealed that DFO significantly inhibited the maturation of BMDC cells in the NP-PF + H group, and all three inhibitors markedly reduced cytokine generation in the NP-PF + H group (Fig. 4E‒G). However, the three ferroptosis inhibitors had no significant effect on BMDC maturation and cytokine secretion in the other treatment groups. Hence, HIFU-induced cell ferroptosis (8.5 W for 30 s, or 3.162 J/mm^3^) had a weak effect on BMDC.

### 3.5 Nano-enhancer enhanced HIFU treatment efficacy on TNBC models

The pharmacokinetics behavior of NP-PF was similar to that of NP, and the addition of PFOB did not affect the pharmacokinetic parameters of NP (Supporting Information Fig. S9A and Table S1). The tissue distribution demonstrated that NP-PF could accumulate at the tumor site of 4T1 tumor-bearing mice (Fig. S9B‒S9D).

Considering the variations in the effectiveness of HIFU treatment throughout the tumor progression, we are interested in determining the optimal timing for HIFU therapy to achieve the best inhibitory effects. Drawing inspiration from the clinical evaluation of tumor suppression effects, which often involves calculating the tumor growth rate (TGR)^41^. We have introduced a hypothetical index, UTGR, to indicate the optimal timing for HIFU treatment. A UTGR value below 0 indicates a stage where the growth rate is reduced, while a UTGR value above 0 suggests an increase in tumor growth rate, indicating the need for timely growth inhibition.

**Figure 5:**
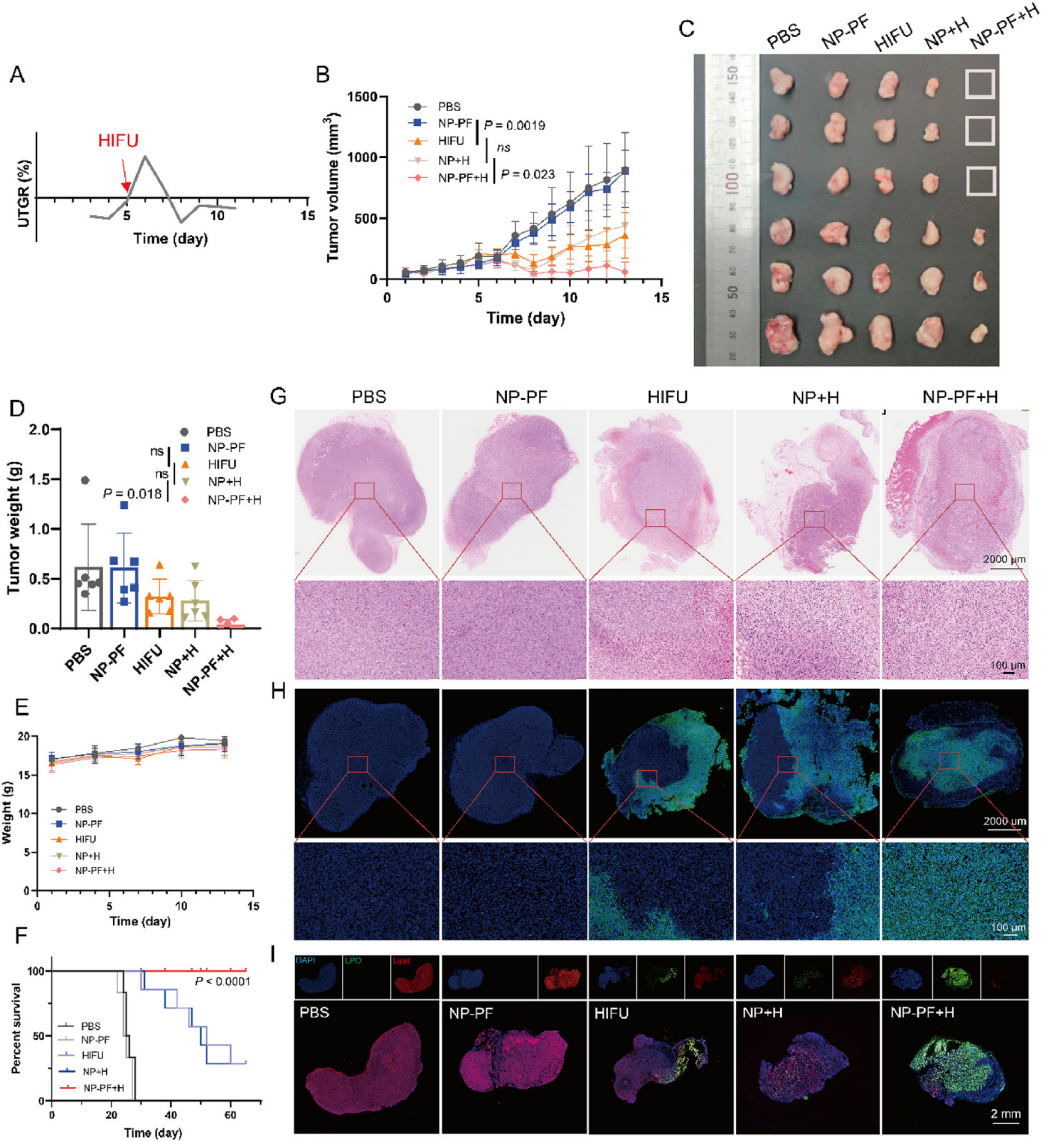
NP-PF enhanced HIFU treatment efficacy on TNBC models. (A) Curve of tumor UTGR change in the PBS group after tumor cell inoculation. The red arrow indicates the time point of HIFU treatment. (B) Tumor growth curves of 4T1-bearing mice after different treatments at UTGR = 0 stages of the tumor (*n* = 6). (C, D) Photograph images (C) and the tumor weight (D) of excised tumors in various treatment groups (*n* = 6). (E) Changes in the body weight of tumor-bearing mice in different treatment groups (*n* = 6). (F) Survival curves of 4T1 tumor-bearing mice from different treatment groups within 65 days (*n* = 6). (G, H) Representative images of tumor slices from different treatment groups after H&E staining (G) and TUNEL staining (H). Scale bar in above = 2000 μm; Scale bar in below = 100 μm. (I) Representative images of tumor slices from different treatment groups after staining with BODIPYTM581/591 C11. Green signals indicated LPO and red signals indicated normal lipids. Scale bar in above = 2 mm. Data are expressed as mean ± SEM.

Next, we applied this indicator (UTGR) in the efficacy evaluation of mice, using 4T1 tumor-bearing mice as the treatment model. We initially recorded the tumor growth curve from the first day of tumor implantation in untreated control mice and calculated the corresponding UTGR (Fig. 5A). Based on the UTGR values, we selected different treatment timings for tumor-bearing mice. When UTGR >0 (Day 7), as observed from the tumor growth curve in Fig. S10, the HIFU treatment group partially inhibited tumor volume to some extent of 66.2% compared with PBS treatment, the tumor still exhibited slow growth. Even when combined with NP-PF, the inhibition of tumor growth by HIFU treatment was not significantly improved. When UTGR = 0 (Day 5), HIFU, NP + H, and NP-PF + H treatments significantly suppressed mouse tumor growth three days post-treatment (Fig. 5B). However, tumors in the HIFU and NP + H groups resumed growth afterward, while tumors in the NP-PF + H group were effectively controlled (Supporting Information Fig. S11). As shown in Fig. 5C and Supporting Information Fig. S11, after 8 days of treatment, NP-PF + H treatment achieved a 50% complete tumor regression, and the average tumor weight of the entire group decreased 20-fold compared to the PBS group (Fig. 5D). In addition, the body weight of mice in the treatment group did not change significantly (Fig. 5E). Survival analysis showed that HIFU, NP + H, and NP-PF + H treatments could significantly prolong the survival time of the model mice compared with PBS or NP-PF treatments. However, due to the tumor recurrence, the survival rate of the mice in the HIFU and NP + H groups within 65 days was 16.7%, while that of the NP-PF + H group was 100% (Fig. 5F). These results indicate the tumor reached a state of growth rate equilibrium when UTGR was 0, at which point HIFU treatment can exert superior antitumor efficacy and reduce the risk of recurrence.

When UTGR was 0, the *in vivo* tumor-killing effect induced by HIFU-triggered ferroptosis was observed through tissue slices. H&E and TUNEL staining results demonstrated that HIFU and NP + H treatments only caused significant cell necrosis and apoptosis around the tumor while NP-PF + H treatment led to a broader destructive effect in the tumor (Fig. 5G and H). This might be due to that NP-PF amplified the penetration and energy effects of HIFU. The level of LPO at the tumor site was detected using the BODIPY probe. Compared to the PBS and NP-PF groups, HIFU and NP + H treatment groups enhanced the expression of LPO to some extent (Fig. 5I), exhibiting weak green fluorescence (LPO) around the tumor periphery. In contrast, the NP-PF + H group showed much stronger overall green fluorescence (LPO) distributed throughout the internal area of the tumor and sharply decreased red fluorescence signals (the normal lipids). Therefore, these results suggest that NP-PF can strengthen the ferroptotic effect of HIFU on tumor cells, resulting in a more effective cytotoxic impact.

### 3.6 NP-PF improved the antitumor immunity of HIFU treatment

**Figure 6:**
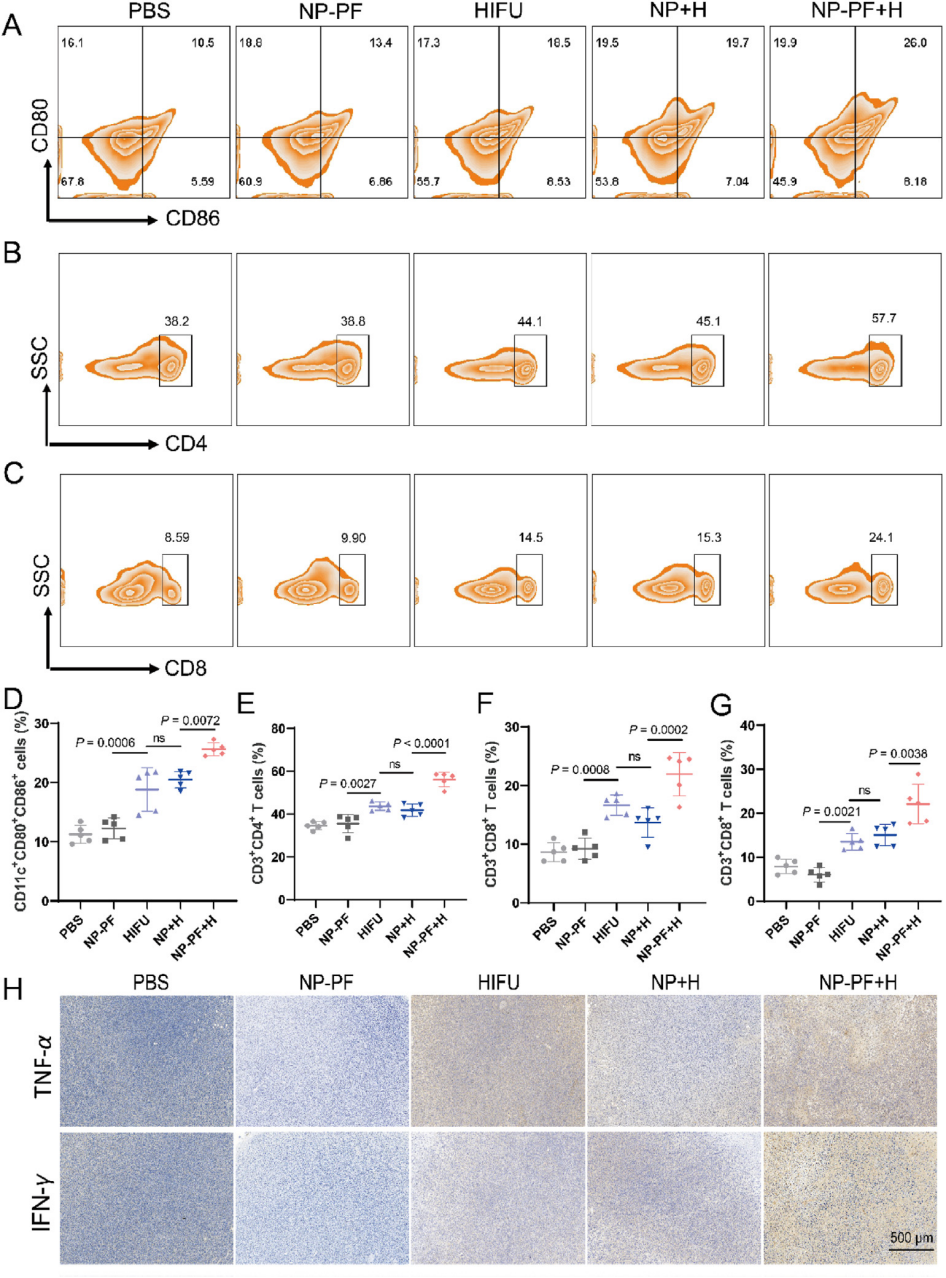
NP-PF improved the antitumor immunity of HIFU treatment. (A–F) Representative flow cytometry charts of mature DCs (A), CD3^+^CD4^+^ T cells (B), and CD3^+^CD8^+^ T cells (C)and the corresponding quantitative results of mature DCs (D), CD3^+^CD4^+^ T cells (E), and CD3^+^CD8^+^ T cells (F) in the tumor-draining lymph nodes from different treatment groups (*n* = 5). (G) The quantitative results of CD3^+^CD8^+^ T cells (F) in tumors from different treatment groups (*n* = 5). (H) Immunohistochemistry staining of TNF-*α* and IFN-*γ* in tumor slices from different treatment groups. Data are expressed as mean ± SEM. Scale bar in above = 500 μm.

The ability of HIFU treatment to trigger BMDC maturation by inducing tumor cell ferroptosis in *in vitro* experiments inspired us to explore the anti-tumor immune response capability of HIFU treatment *in vivo*. When the tumor volume reached 100 mm^3^, the mice were randomly divided into five groups and received different treatments. On the 7th day after treatment, tumors, tumor-draining lymph nodes (TDLN), and spleens were harvested and immune cells were analyzed. It was found HIFU treatment demonstrated a notable capacity to increase the proportion of mature DC (CD11c^+^CD80^+^CD86^+^) and the percentage of CD3^+^CD4^+^ and CD3^+^CD8^+^ T cells in the TDLN (Fig. 6A‒F). Notably, NP-PF significantly amplified the activation of both DCs and T cells induced by HIFU treatment, while NP did not exhibit this enhancing effect. Subsequently, we explored the immune microenvironment at the tumor site, where CD8^+^ T cells, as the main force of adaptive immune responses, play a crucial role in tumor destruction^42^. In the PBS and NP-PF groups, the proportion of CD3^+^CD4^+^ T cells was less than 5%, and the percentage of CD8^+^ T cells was below 10%. The HIFU group showed a significant increase in T cell proportions, while the NP-PF + H group exhibited the highest percentages of CD3^+^CD4^+^ T cells and CD3^+^CD8^+^ T cells, approximately 4.6 times and 2.8 times higher than the PBS group, respectively (Fig. 6G and Supporting Information Fig. S12). At the same time, the immunohistochemical staining of tumor slices demonstrated that HIFU could upregulate the expressions of TNF-*α* and IFN-*γ* in tumors and those of the NP-PF + H group were the highest among all the groups (Fig. 6H).

**Figure 7:**
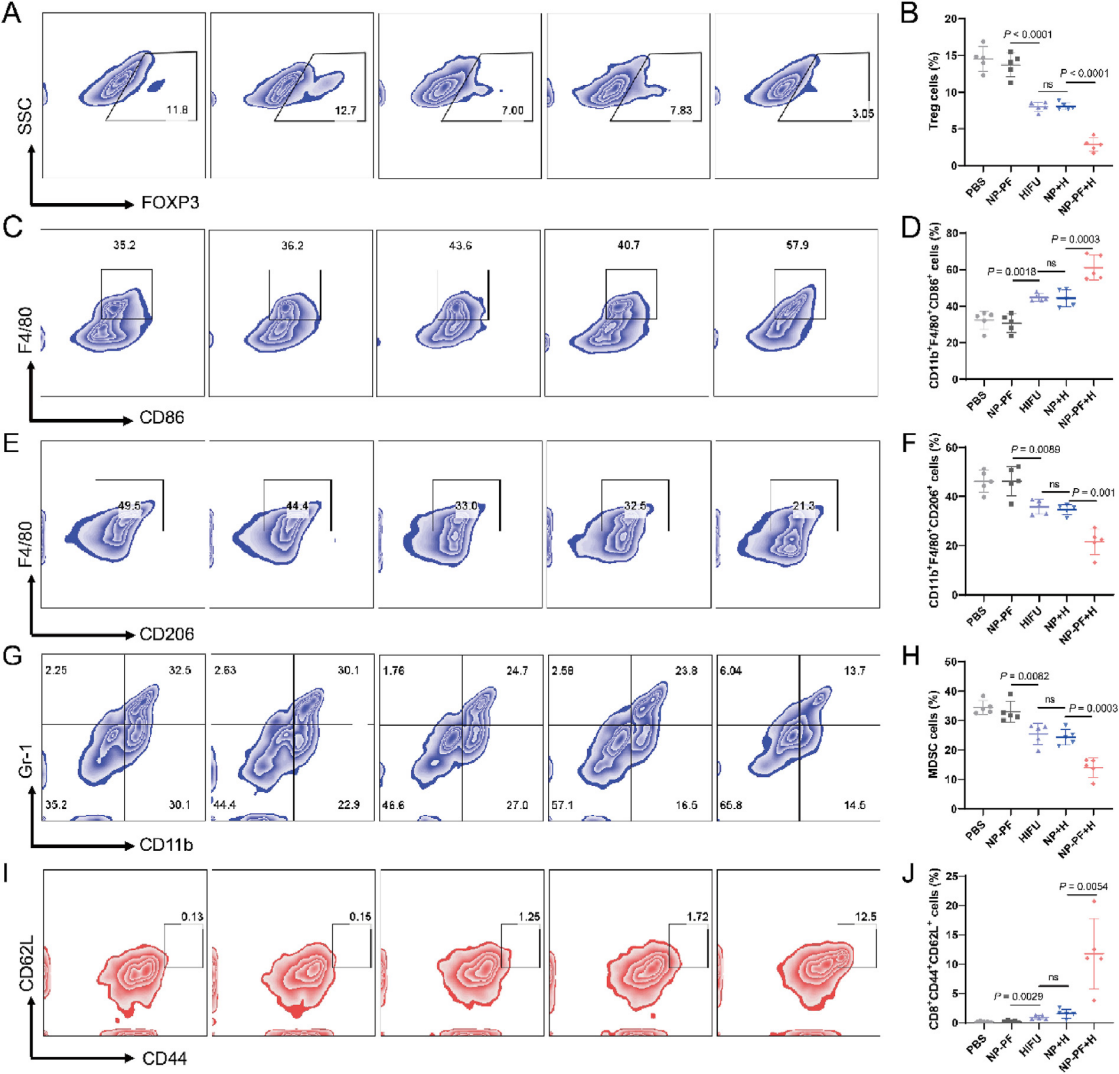
Reversal of tumor immune suppressive microenvironment by NP-PF + H treatment. (A, B) Representative flow cytometry charts (A) and the corresponding quantitative results (B) of Treg cells in tumors from different treatment groups (*n* = 5). (C–F) Representative flow cytometry charts of M1-type (C) and M2-type macrophages (E) and the corresponding results of M1-type (D) and M2-type macrophages (F) in tumors from different treatment groups (*n* = 5). (G, H) Representative flow cytometry charts (G) and the corresponding quantitative results (H) of MDSC cells in tumors from different treatment groups (*n* = 5). (I, J) Representative flow cytometry charts (I) and the corresponding quantitative results (J) of CD44^+^CD62L^+^ memory T cells in the spleens from different treatment groups. Data are expressed as mean ± SEM.

The low T cell infiltration in the tumor is mainly caused by the inhibitory tumor immune microenvironment^43^. Thus, we investigated the changes in immune suppressive cells in tumors after different treatments. As shown in Fig. 7, the tumor of the PBS group had a high proportion of Treg cells (14.5%) and MDSC cells (34.5%), with Treg cells outnumbering CD8^+^ T cells (Fig. 7A, B, G, H). HIFU treatment significantly reduced the proportions of these cells, and NP + H treatment showed similar effects to HIFU treatment. Interestingly, NP-PF + H treatment achieved the maximum reduction, bringing Treg and MDSC cells down to 2.8% and 14.0%, respectively. M1-type tumor-associated macrophages can secrete pro-inflammatory cytokines with killing effects to benefit anti-tumor immune function, while M2-type tumor-associated macrophages can help tumor cells escape from killing, and the two types can switch to each other with the change of tumor microenvironment^44^. HIFU, NP + H, and NP-PF + H treatments significantly increased the proportion of M1-type macrophages and decreased the proportion of M2-type macrophages. Importantly, NP-PF + H treatment could maximize the proportion of M1-type cells from 32.4% to 61.0% and decrease the proportion of M2-type cells from 46.2% to 21.5% compared to PBS treatment (Fig. 7C‒F). In order to further explore whether the mice after HIFU treatment could produce immune memory to prevent tumor recurrence, we detected the number of central memory T cells in the spleen. Compared with the PBS group, the proportion of CD44^+^CD62L^+^ memory T cells in the HIFU and NP + H groups only increased by 1% (Fig. 7I and J) and NP-PF + H triggered the highest percentage of central memory T cells (more than 10%) among all treatment groups. In conclusion, NP-PF + H treatment could activate the adaptive immune response and reverse the “cold” tumor environment to a therapy-friendly “hot” tumor environment, thereby killing tumors more effectively and harvesting immune memory to prevent tumor recurrence.

### 3.7 Safety assessment of NP-PF

**Figure 8:**
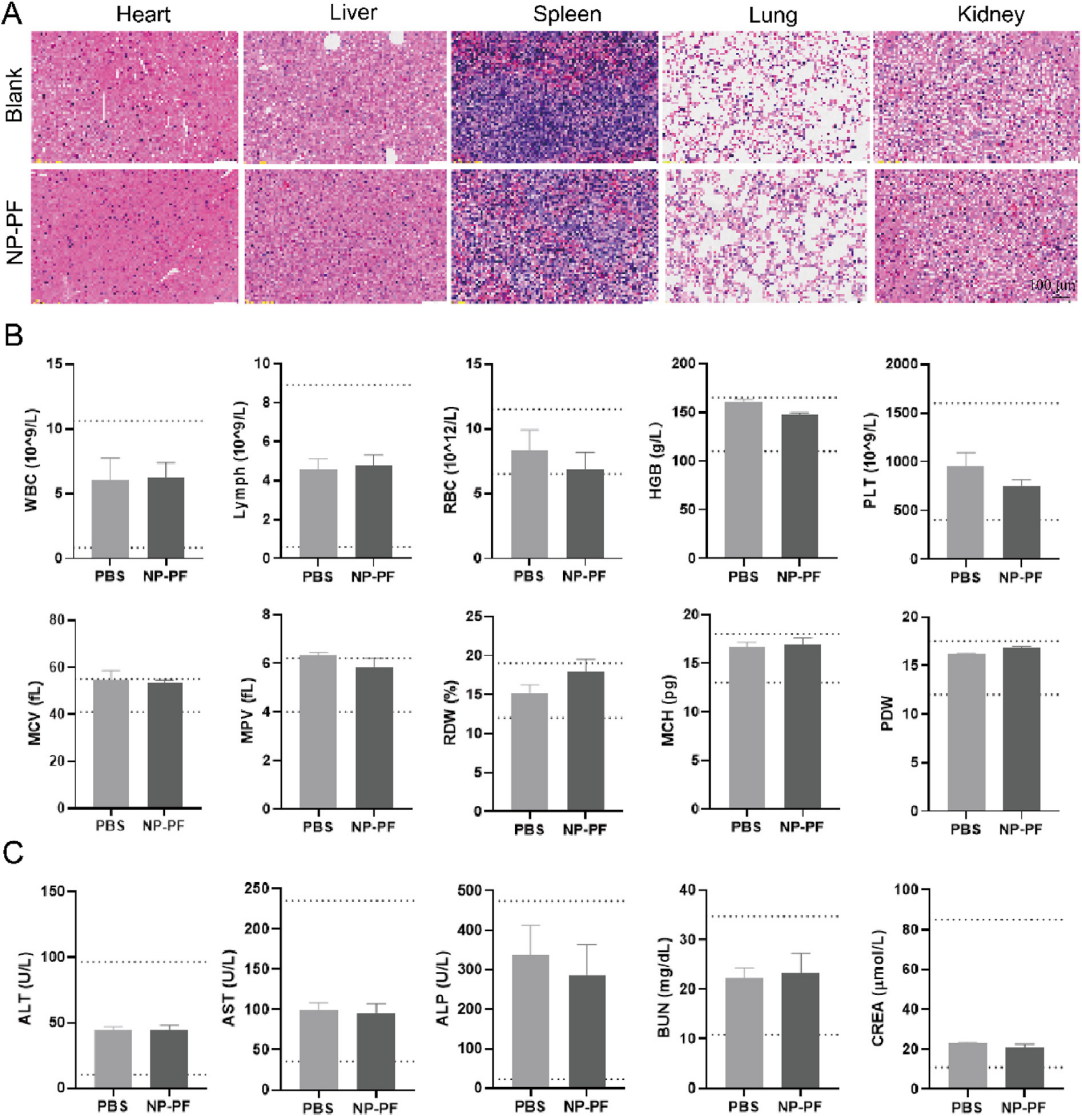
Safety assessment of NP-PF. (A) Representative images of H&E-stained slices of major organs from mice treated with NP-PF or PBS. (B) Blood routine data in 4T1 tumor-bearing BALB/c mice after PBS or NP-PF treatment (*n* = 3). (C) Blood biochemistry data in 4T1 tumor-bearing BALB/c mice after PBS and NP-PF treatments (*n* = 3). Data were expressed as mean ± SEM. Scale bar above = 100 μm.

We then evaluated the *in vivo* safety of the nano-enhancer NP-PF through intravenously injecting the therapeutic dose of NP-PF into mice. After 13 days compared with the PBS group, NP-PF did not cause any damage to the heart, liver, spleen, lung, or kidneys (Fig. 8A). Moreover, blood routine-related indicators of the NP-PF group were within normal ranges (Fig. 8B). Blood biochemical results revealed the NP-PF group had normal liver function indicators including aspartate aminotransferase (AST), alkaline phosphatase (ALP), and alanine transaminase (ALT), and normal kidney function indicators including creatinine (CRE) and blood urea nitrogen (BUN) (Fig. 8C). In conclusion, NP-PF was a safe and biocompatible nano-enhancer.

## 4 Discussion

As a non-invasive treatment technique, HIFU can be used not only directly for clinical tumor ablation but also in conjunction with drug delivery systems for HIFU-responsive drug release. In clinical practice, the ablative effect of HIFU for tumor destruction is mainly enhanced through microbubbles, and this effect is suitable for various solid tumors. However, the limited tissue penetration of sound waves determines the differences in the therapeutic efficacy of tumors at different stages. Therefore, choosing the right timing for HIFU treatment is crucial. The parameter UTGR introduced in our study provides guidance on the treatment timing. When choosing UTGR >0 for HIFU treatment, the tumor ablation is incomplete and prone to recurrence. If choosing a UTGR value of 0 for HIFU treatment, combined with the nano-enhancer, a high even complete tumor suppression can be achieved. We hypothesize that nano-ehancer-assisted HIFU therapy during the early stages of tumor growth, where UTGR <0, might potentially yield greater benefits for patients by achieving complete tumor ablation. However, if the tumor is in the later stages of growth with UTGR <0, the large tumor volume may reduce the benefits derived from HIFU treatment for the patients. Whether multiple HIFU treatments can be applied to recurrent tumors and determining the corresponding optimal treatment nodes based on UTGR are still worth further exploring.

The use of ultrasound-assisted drug delivery plays a crucial role in the field of cancer treatment, with numerous studies currently underway. Since microbubbles were approved by the FDA for clinical use as ultrasound contrast agents, they have been widely studied in the field of drug delivery. Related research tends to use HIFU as a responsive stimulus, combined with drug delivery systems for drug release, gene expression, etc^45^^,^^46^. However, the mechanism of HIFU and microbubbles on cells is often overlooked while focusing on the drug's action. The known enhancement mechanism of microbubbles is primarily based on the cavitation effect, which is the response to pressure wave oscillation caused by their compressible core. As the most crucial ultrasound parameter, acoustic power determines the different responses of microbubbles: stable cavitation occurs at low acoustic pressure, while inertial cavitation occurs at high acoustic pressure^47^. In our study, we have established that HIFU can induce ferroptosis in tumor cells and the combined application of NP-PF can significantly enhance the ferroptotic damage of HIFU with a specific EEF (*e.g.*, EEF = 3.162 J/mm^3^) to tumor cells. Exploring the impact of different cavitation effects produced by microbubbles under HIFU stimulation at different acoustic powers on the ferroptotic effects triggered by HIFU is a valuable aspect that warrants further investigation.

The EEF can serve as a quantitative metric reflecting the level of HIFU treatment on tumors. In this study, we have only conducted a preliminary exploration of the relationship between EEF and ferroptosis, as well as the level of LPO. We found that this indicator could assist in identifying the interaction between HIFU and cell death efficiency, guiding the selection of optimal parameters to induce ferroptosis. The indication provided by the HIFU-triggered ferroptosis-immunogenic effect suggests the possibility of combining other anti-tumor drugs, such as ferroptosis inducers or immunotherapeutic agents, for more effective ablation treatment in clinically advanced-stage cancer patients.

## 5 Conclusions

In summary, we validated that HIFU with different technical parameters could trigger ferroptosis *via* mechanochemical disruption of the GSH/GSSG balance, and revealed that combining HIFU with nano-enhancers could enhance the ferroptosis effect in tumor cells. This transformation of the “cold” tumor microenvironment of triple-negative breast cancer into a “hot” tumor environment resulted in an improved anti-tumor immune response. For triple-negative breast cancer cell-bearing mice, intravenous injection of NP-PF followed by HIFU treatment at the stage of UTGR = 0 led to a superior tumor suppression rate and triggered an enhanced anti-tumor immune response *in vivo*. Overall, this provides valuable guidance for the clinical application of HIFU in tumor immunotherapy.

## Author contributions

Conceptualization: Xuejing Li and Zhiqing Pang; Methodology: Xuejing Li and Zhiqing Pang; Investigation: Xuejing Li, Jiayi Wu, Ruizhe Xu, Xifeng Qin, Siyu Wang and Wuli Yang; Funding acquisition: Zhiqing Pang; Supervision: All authors; Writing—original draft: Xuejing Li and Zhiqing Pang; Writing—review and editing: All authors.

## Conflicts of interest

The authors declare that they have no competing financial interests.

## Appendix A Supplementary data

The following is the Supporting Information to this article:
