# Supplementary material for: High intensity forced ultrasound-driven ferroptosis as a strategy for anti-tumor immune priming
Source: Acta Pharm Sin B. 2025 May 28;15(7):3788–804. doi: 10.1016/j.apsb.2025.05.006 (PMC12278624; doi:10.1016/j.apsb.2025.05.006)
Supplement: Multimedia component 1 [file mmc1.pdf]

Supporting Information
ORIGINAL ARTICLE

High intensity forced ultrasound-driven ferroptosis as a strategy for anti-tumor immune priming

Xuejing Li[a], Jiayi Wu[a], Ruizhe Xu[a], Xifeng Qin[a], Siyu Wang[a], Wuli Yang[b], Zhiqing Pang[a,*]

[a]*Key Laboratory of Smart Drug Delivery, School of Pharmacy, Fudan University, Shanghai 201203, China*

[b]*State Key Laboratory of Molecular Engineering of Polymers & Department of Macromolecular Science, Fudan University, Shanghai 200433, China*

Received 20 November 2024; received in revised form 12 February 2025; accepted 13 March 2025

*Corresponding author.

E-mail address: zqpang@fudan.edu.cn (Zhiqing Pang).

Methods

1. Pharmacokinetics study of NF-NP

DiD labeled NP-PF or NP was prepared with the same method as NP-PF or NP except that 1 μg of DiD was added into the organic phase. When the tumor volume of 4T1 tumor-bearing mice reached around 100 mm^3, 150 μL of DiD-NP or DiD-NP-PF were injected intravenously at the DiD dose of 2 μg, respectively, and 50 μL of blood was taken from the orbit of mice at preset time points and mixed with 50 μL of EDTA-2Na solution. Subsequently, the fluorescence intensity of the blood samples was determined using a microplate reader (Ex/Em=640/670nm, Tecan, Switzerland).

2. Tissue distribution of NF-NP

The 4T1-bearing mice were randomly divided into two groups. When the tumor volume grew to around 100 mm^3, PBS and DiD-NP-PF (the DiD dose of 2 μg) were injected into the tail vein, respectively, and the mice were imaged in the IVIS Spectrum live-imaging system (PerkinElmer, USA) 24 hours later. Major tissues and tumors were then dissected and imaged *ex vivo* (PerkinElmer, USA). After imaging, the tissues and tumors were collected and homogenized and the fluorescence intensity was detected by a microplate reader (Ex/Em=640/670nm, Tecan, Switzerland). The concentration of NP-PF in each organ was calculated and expressed as ID% (Percentage of injected dose).

Results

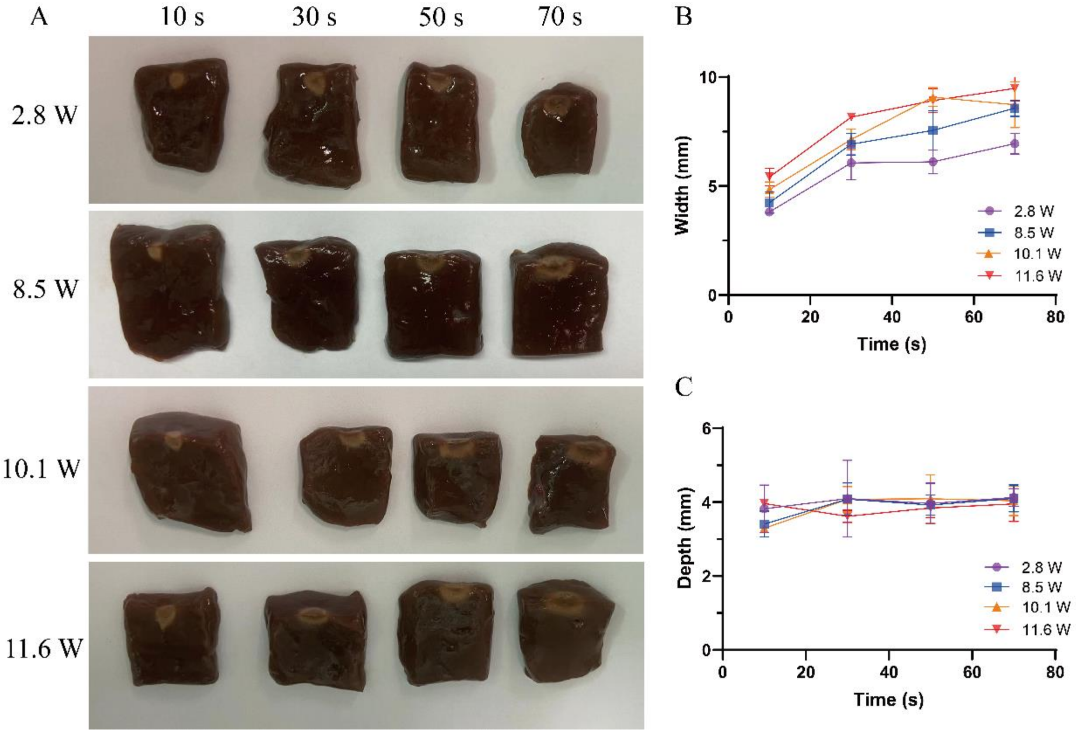

Figure S1. (A) Representative photographs of pork livers cut along the direction of HIFU irradiation after different power. (B, C) The radiation width (B) and depth (C) of pork livers irradiated with HIFU at different power (n=3).

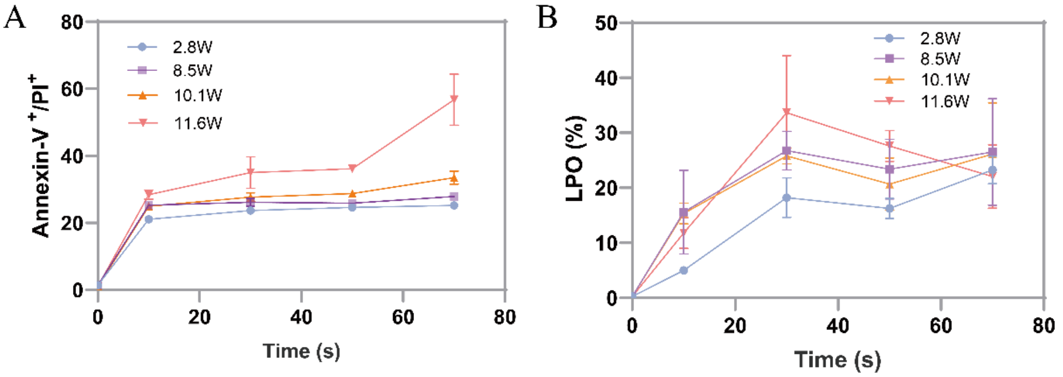

Figure S2. Apoptosis level (A) and LPO level (B) in 4T1 cells after HIFU treatment with different energies (n=3).

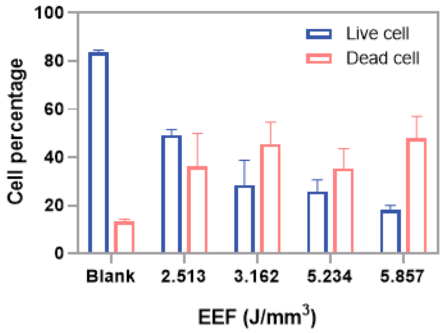

Figure S3. The live/dead rate of 4T1 cells after HIFU treatment with different energy efficiency factors (n=3).

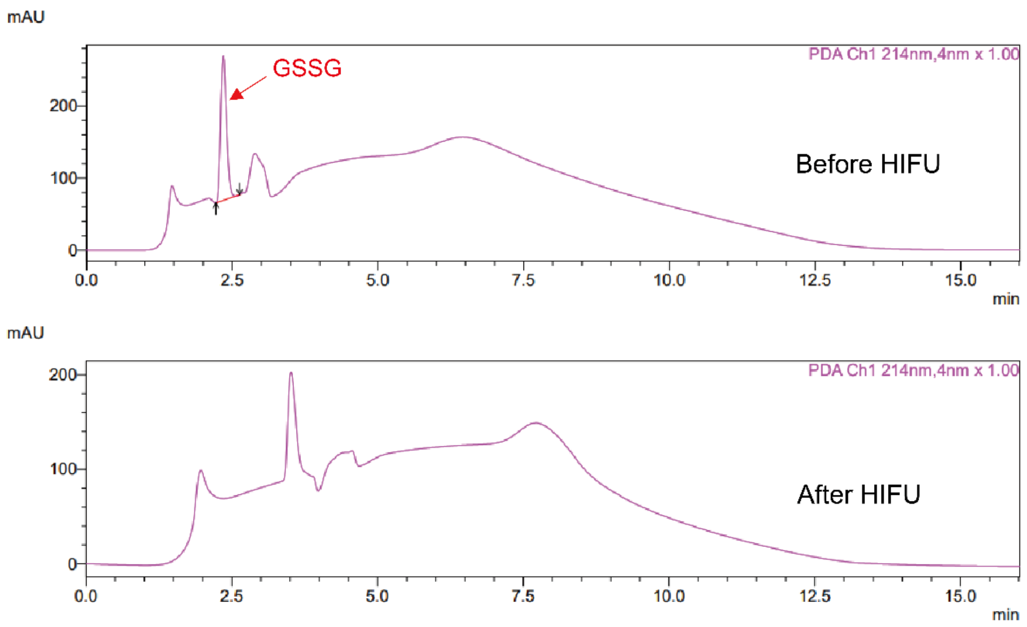

Figure S4. Representative HPLC images of GSSG before and after HIFU treatment.

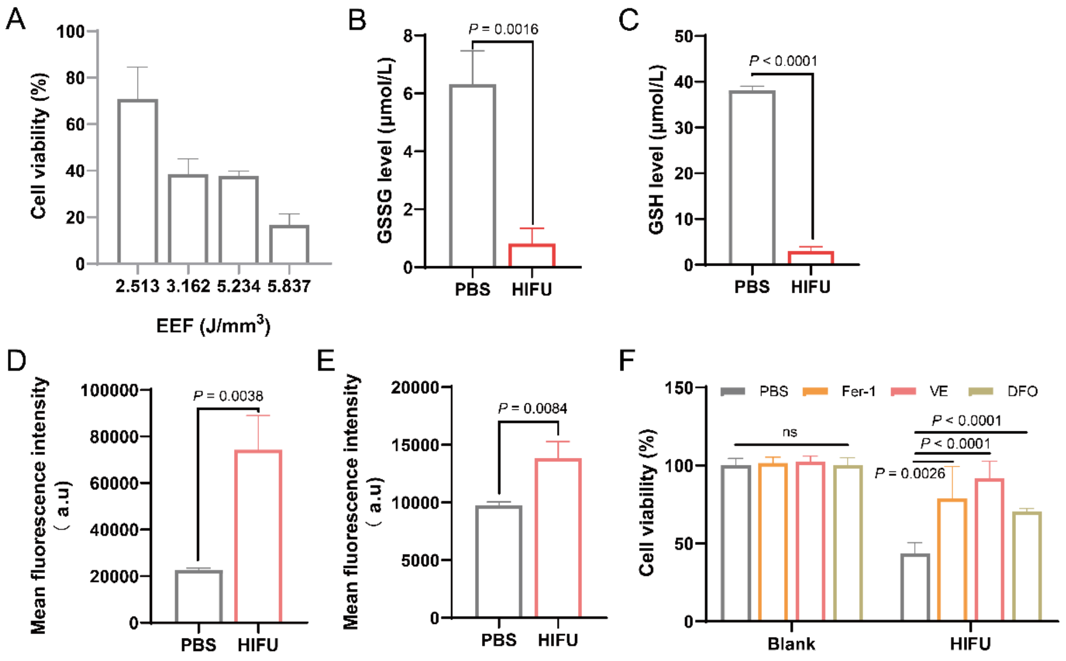

Figure S5. HIFU-induced ferroptosis in CT-26 cells. (A) The cell viability of CT-26 cells treated with HIFU with different EEF values (n=3). (B) The GSSG content in CT-26 cells treated with HIFU at the EEF value of 5.234 J/mm^3 (n=3). (C, D) The GSH content (C) and ROS content (D) in CT-26 cells treated with HIFU at the EEF value of 5.234 J/mm^3 (n=3). (E) Lipid peroxidation (LPO) level in CT-26 cells treated with HIFU at the EEF value of 5.234 J/mm^3 (n=3). (F) The cell viability of CT-26

cells that received HIFU treatment (at the EEF value of 5.234 J/mm^3) in the presence of different ferroptosis inhibitors including Fer-1, DFO, and VE (n=3). Data were expressed as mean ± SEM.

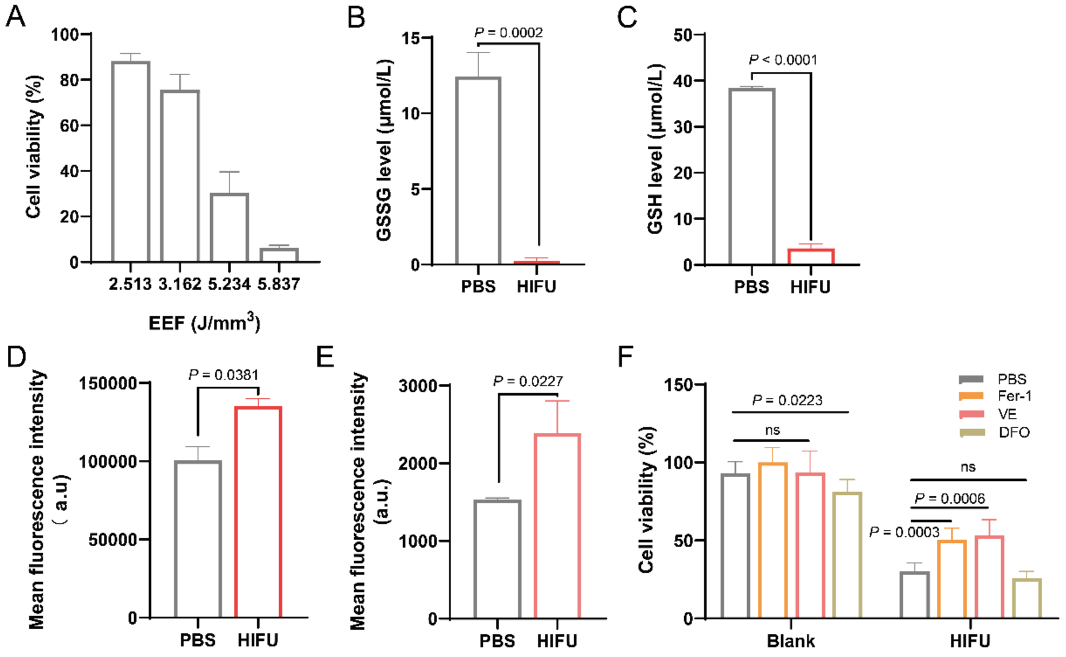

Figure S6. HIFU-induced ferroptosis in TC-1 cells. (A) The cell viability of TC-1 cells treated with HIFU with different EEF values (n=3). (B) The GSSG content in TC-1 cells treated with HIFU at the EEF value of 5.234 J/mm^3 (n=3). (C, D) The GSH content (C) and ROS content (D) in TC-1 cells treated with HIFU at the EEF value of 5.234 J/mm^3 (n=3). (E) Lipid peroxidation (LPO) level in TC-1 cells treated with HIFU at the EEF value of 5.234 J/mm^3 (n=3). (F) The cell viability of TC-1 cells that received HIFU treatment (at the EEF value of 5.234 J/mm^3) in the presence of different ferroptosis inhibitors including Fer-1, DFO, and VE (n=3). Data were expressed as mean ± SEM.

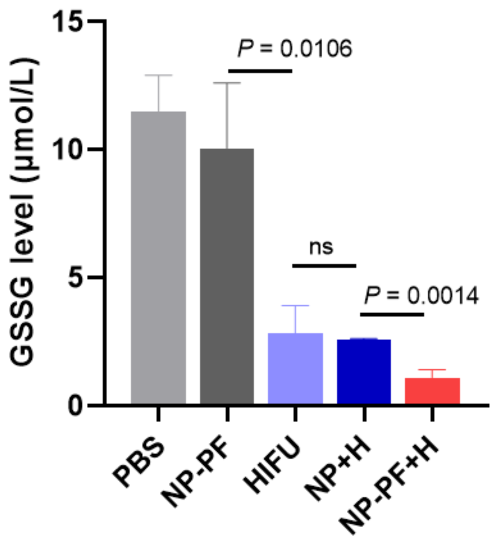

Figure S7. Intracellular GSSG content in 4T1 cells after different treatments (n=3).

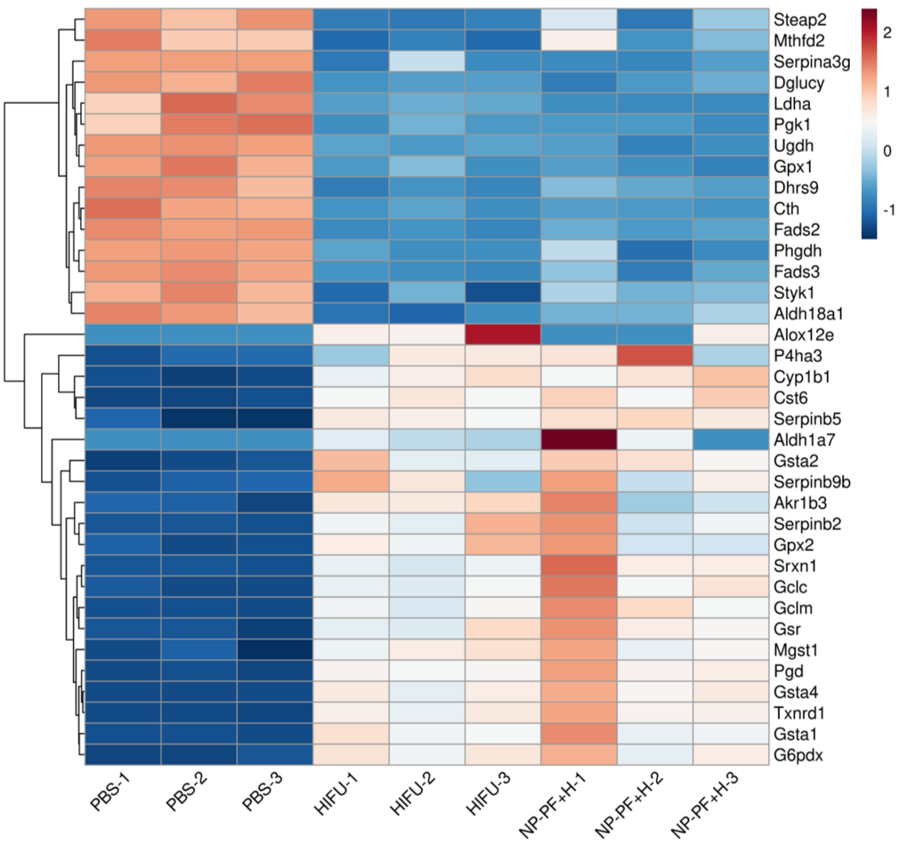

Figure S8. Heat map of differential gene expression involved in redox processes in 4T1 cells after different treatments (n=3). The number in the scale plate referred to the log2fold change.

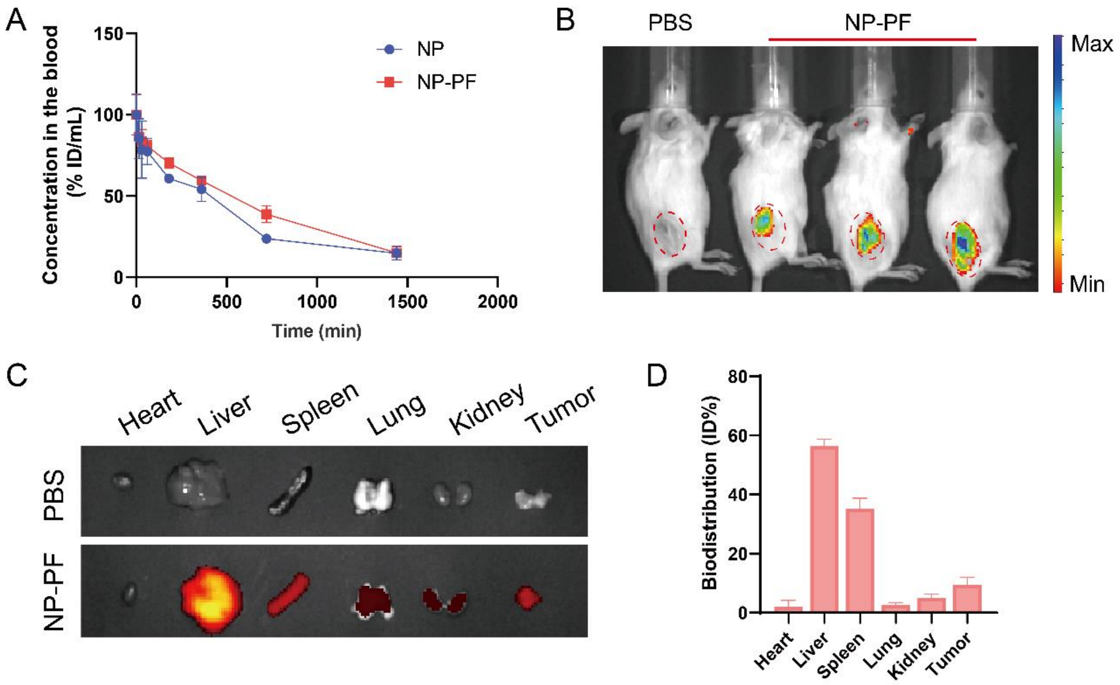

Figure S9. (A) Pharmacokinetic curves of NP and NP-PF in 4T1 tumor-bearing mice. (B) Representative *in vivo* fluorescence images of 4T1 tumor-bearing mice at 24 h after intravenous injection of DiD-labeled NP-G/P. (C) Representative *ex vivo* imaging of major organs and tumors at 24 h after injection. (D) Quantitative biodistribution of NP-PF in major organs and tumors at 24 h after injection (n = 3).

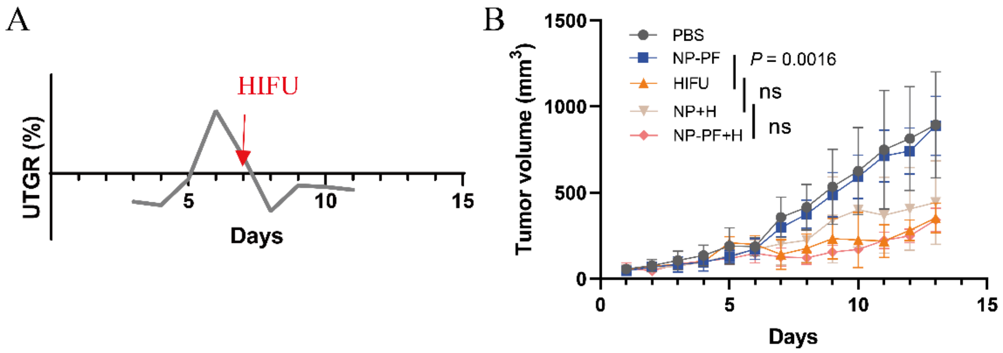

Figure S10. (A) The curve of tumor UTGR change in the PBS group after tumor cell inoculation. The red arrow indicates the time point of HIFU treatment (day 7). (B)Tumor growth curves of 4T1-bearing mice after different treatments at the UTGR > 0 stage (n=6).

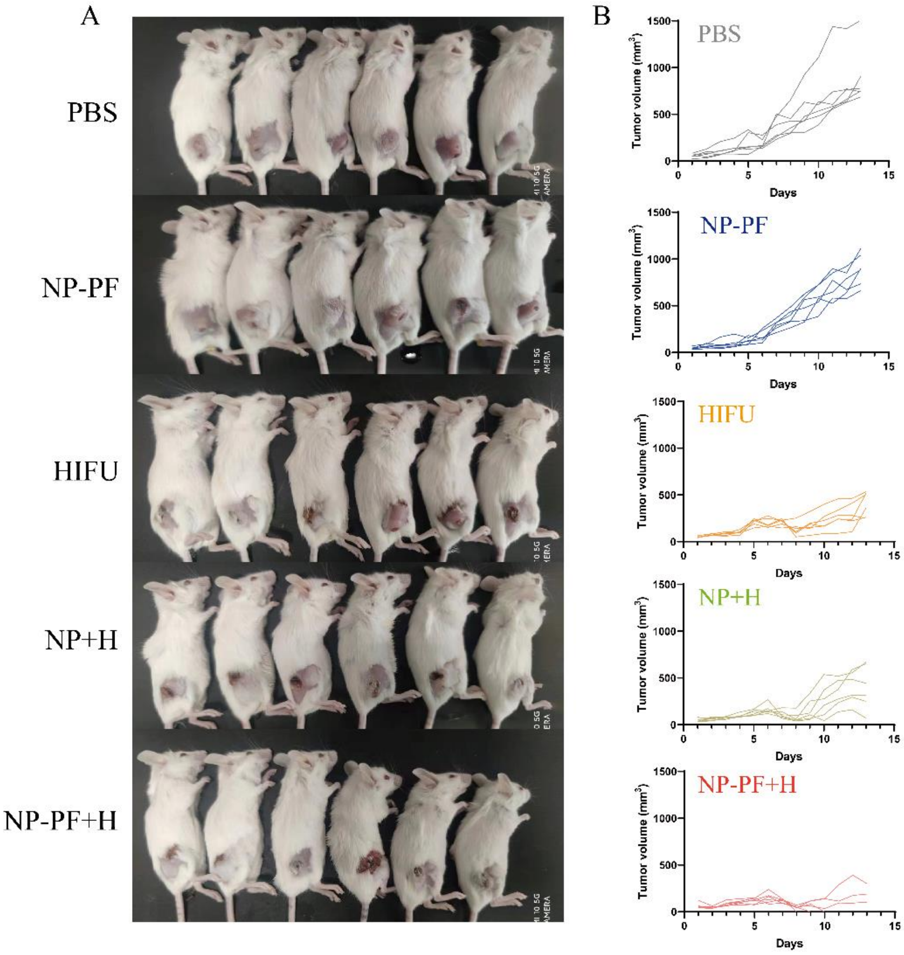

Figure S11. (A) Photographs of primary tumors in the different treatment groups on day 13 (n=6). (B) Tumor growth curves of primary tumors from individual mice in different treatment groups (n=6).

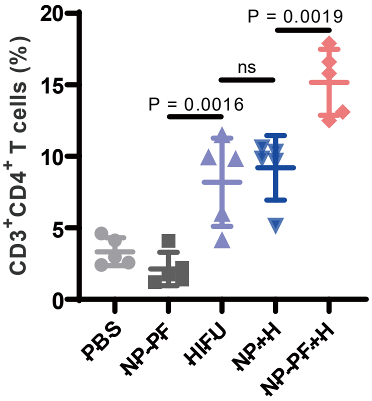

Figure S12. The quantitative results of $CD3^{+}CD4^{+}$ T cells from the tumors after different treatments (n=5).

Table S1. Pharmacokinetic parameters of NP and NP-GF in 4T1-tumor-bearing mice

Parameters	NP	NP-GF
$t_{1/2}$ (h)	7.86±0.15	9.21±1.06
$AUC_{0-\infty}$ (%ID/mL*h)	1019.05±48.75	1243.30±122.75
CL(L/h/kg)	0.09±0.005	0.08±0.008
